# Alterations in Glutathione Redox Homeostasis in Metabolic Dysfunction-Associated Fatty Liver Disease: A Systematic Review

**DOI:** 10.3390/antiox13121461

**Published:** 2024-11-28

**Authors:** Lucia Cesarini, Flavia Grignaffini, Anna Alisi, Anna Pastore

**Affiliations:** Research Unit of Genetics of Complex Phenotypes, Bambino Gesù Children’s Hospital, IRCCS, 00165 Rome, Italy; lucia.cesarini@opbg.net (L.C.); flavia.grignaffini@uniroma1.it (F.G.); anna.pastore@opbg.net (A.P.)

**Keywords:** MASLD, GSH, glutathione, low molecular weight thiols

## Abstract

Low molecular weight (LMW) thiols, particularly glutathione, play pathogenic roles in various multiorgan diseases. The liver is central for the production and systemic distribution of LMW thiols; thus, it is particularly susceptible to the imbalance of redox status that may determine increased oxidative stress and trigger the liver damage observed in metabolic dysfunction-associated steatotic liver disease (MASLD) models and humans. Indeed, increased LMW thiols at the cellular and extracellular levels may be associated with the severity of MASLD. Here, we present a systematic literature review of recent studies assessing the levels of LMW thiols in MASLD in in vivo and in vitro models and human subjects. Based on the PRISMA 2020 criteria, a search was conducted using PubMed and Scopus by applying inclusion/exclusion filters. The initial search returned 1012 documents, from which 165 eligible studies were selected, further described, and qualitatively analysed. Of these studies, most focused on animal and cellular models, while a minority used human fluids. The analysis of these studies revealed heterogeneity in the methods of sample processing and measurement of LMW thiol levels, which hinder cut-off values for diagnostic use. Standardisation of the analysis and measure of LMW thiol is necessary to facilitate future studies.

## 1. Introduction

Low-molecular-weight (LMW) thiols, including glutathione (GSH), cysteine (Cys), cysteinyl-glycine (CysGly), and homocysteine (Hcy), are ubiquitous molecules that exert several biological functions. Still, their pivotal role is to preserve redox homeostasis in the cell [1,2]. In particular, LMW thiols may regulate the activity of specific antioxidant enzymes by acting as cofactors and several proteins by establishing covalent bonds with them, thus protecting the cell from oxidative stress [1]. Moreover, LMW thiols may directly donate electrons to oxidants, form complexes with metal ions, and bind xenobiotic agents, thus helping cells in detoxification [1].

Among the LMW thiols, GSH, a tripeptide composed of sequential Glu, Cys, and Gly residues, represents the first line of cellular defence against oxidative stress. Intracellular levels of GSH range from 1 to 15 mM in the cytoplasm and from 10 to 14 mM in mitochondria [3]. In particular, in cultured cells, GSH values were reported to range approximately from 20 to 150 nmol/mg of proteins, whereas the extracellular levels of GSH range between 2 and 20 µM, values often found in total body fluids. Approximately 99% of the intracellular GSH was found in a reduced (GSH) state and 1% in the oxidized form (GSSG). At the same time, different values of extracellular GSH/GSSG ratios were reported in various body fluids [4]. These latter values could represent the benchmark for assessing GSH and GSSG amounts as biomarkers in different pathological conditions, including neurological disorders, type 2 diabetes, and cardiovascular and liver diseases [5,6,7,8]. However, despite heterogeneity in the most appropriate methods for sample pre-treatment and quantification, there is a strong interest in using the GSH/GSSG ratio to assess redox status. Therefore, reported concentrations can vary widely across laboratories [9]. Measurement of GSH and GSSG may be performed by using different approaches (e.g., Ellman’s method unmodified/modified by using an enzymatic recycling procedure and fluorometric and spectrophotometric assays), even though high-performance liquid chromatography (HPLC) coupled with UV or fluorescent detection, and the addition of N-ethylmaleimide earlier during sample preparation to prevent GSH oxidation is also often used [10,11,12,13].

GSH is mainly produced by the liver, which retains the unique ability to synthesise its precursor (i.e., Cys) and provide the principal reservoir for releasing GSH into circulation [6,14]. Besides its role as a GSH precursor, Cys is central to sulphur metabolism, involving Hcy and serine in the synthesis of GSH. Furthermore, GSH serves as a steady source of Cys through the extracellular degradation to form CysGly via the γ-glutamyl cycle, thus playing a pivotal role in regulating the cellular stress response when Cys levels are low [15]. The total extracellular concentration of free Cys is typically maintained at values ranging from 200 to 300 μM [16], while a healthy Hcy level is generally below 14 μM [17]. A large amount of the literature highlighted that different methods, such as fluorescence or electrochemical detection, may be used to detect Cys, CysGly, and Hcy. HPLC remains the most sensitive approach for simultaneously measuring single thiols, at least in body fluids [18].

Several lines of evidence demonstrated that thiol levels were strongly associated with the onset and progression of different pathologies, with the liver playing a pivotal role in controlling the production of Cys, the limiting molecule for GSH synthesis. Therefore, it is unsurprising that LMW thiols may indicate oxidative stress and are pathogenically involved in a multi-organ disease such as non-alcoholic fatty liver disease (NAFLD) [6]. NAFLD term was recently replaced by metabolic dysfunction-associated steatotic liver disease (MASLD), thus including the entire spectrum of liver damages (i.e., metabolic dysfunction-associated steatohepatitis—MASH, and fibrosis) and metabolic derangements associated with this multi-spectrum disease and avoiding the “fatty liver” stigma [19,20]. MASLD, with an estimated global prevalence of around 30% in adults and around 13% in children, represents the most prevalent chronic liver disease and the principal cause of cirrhosis, hepatocellular carcinoma, hepatic-related mortality, and liver transplantation [20,21]. The multifactoriality of MASLD development and progression may be mainly ascribed to a complex network of molecular events that include genetic background, epigenetic mechanisms, gut dysbiosis, lipid dysmetabolism, insulin resistance, inflammation, and oxidative stress [22,23,24].

Studies in experimental in vitro and in vivo models and humans demonstrated that different molecules and proteins implied in the control of the redox status in the liver cells exhibited a causal link with exacerbation of hepato-metabolic damage occurring in MASLD, MASH and its related fibrosis [25]. The role of thiols was also widely investigated with a primary focus on GSH and Hcy, suggesting that changes in these molecules at cellular and extracellular levels (e.g., blood, blood cells, serum, and plasma) may be associated with the disease severity. However, a literature review of the results addressing the amount of LMW thiols in MASLD by considering experimental studies on in vivo and in vitro models and humans is still lacking. As the potential diagnostic and therapeutic role of these thiols in MASLD, with our systematic review, we aimed to fill the gap by providing a summary of data, methods, and statistical significance of studies assessing the amount of LMW thiols in the disease.

## 2. Materials and Methods

### 2.1. Search Strategy

The present systematic review followed the guidelines for Preferred Reporting Items for Systematic Reviews and Meta-Analyses (PRISMA) [26]. The protocol for the present systematic review was registered on INPLASY (protocol number: INPLASY2024100096).

The search was performed on PubMed, Embase, and Scopus databases for articles published between 1 January 2019 and 30 June 2024. The following advanced search approach was used for PubMed and Embase: (glutathione OR GSH OR thiols) AND (NAFLD) were searched first, then (glutathione OR GSH OR thiols) AND (MAFLD OR MASLD). English language restriction was applied. The following advanced search approach was used for Scopus: TITLE-ABS-KEY ((glutathione OR GSH OR thiols) AND (NAFLD)) were searched first, then TITLE-ABS-KEY ((glutathione OR GSH OR thiols) AND (MAFLD OR MASLD)). The subject area was limited to the “English” language. Articles retrieved by PubMed and Scopus were then merged. Articles retrieved by PubMed, Scopus, and Embase were subsequently merged.

### 2.2. Article Screening and Selection

Duplicate records from the database were removed before the first eligibility screening. Next, we excluded all non-peer-reviewed studies (e.g., conference abstracts, dissertations, and other grey literature). Reviews and articles outside the time window were also excluded.

Overall, the articles publishing data describing alterations in GSH redox homeostasis in the presence of MASLD/NAFLD in animal models, cell lines, and human samples were all considered. In particular, articles were screened by title and abstract to identify those relevant to the topic covered in the present review. However, some articles were excluded as GSH, thiols, or oxidative stress were not mentioned in the abstract and/or title. If the abstract mentioned oxidative stress without mentioning GSH/thiols explicitly, the linked article was further screened for the keywords “GSH”, “glutathione”, “thiol”, and “cysteine*” to check whether the text contained data not described explicitly in the abstract. Articles that assessed oxidative stress or GSH metabolism parameters but did not measure GSH, Cys, CysGly, and Hcy were excluded. Articles mentioning GSH and/or thiols as a diet or treatment component were also excluded.

Moreover, the full text of the articles was screened according to the following objective exclusion criteria: absence of explicit values for LMW thiols, missing methods, missing units of measurement, and missing information on the study design. The examination and screening of all the search results were conducted by three independent authors (L.C., A.A., and A.P.).

### 2.3. Data Extraction

The data extracted from each article were different for experimental models and studies.

Data on the type of the organism (e.g., mice and rats), disease model, length of study, assay methods, biological matrix, and measurements and statistical significance of the amount of GSH (GSH, total glutathione (tGSH), and GSSG) and thiols (Cys, CysGly, and Hcy) were extracted in experimental studies on animal models.

Cellular line type, study length, assay methods, measurements, and statistical significance of the amount of GSH (GSH, tGSH, and GSSG) and thiols (Cys, CysGly, and Hcy) were extracted from experimental studies on cell models.

In human studies, study type, age, number of subjects, assay methods, measurements, and statistical significance of the amount of GSH (GSH, tGSH, and GSSG) and thiols (Cys, CysGly, and Hcy) were captured.

## 3. Results

### 3.1. Search Results

**Figure 1:**
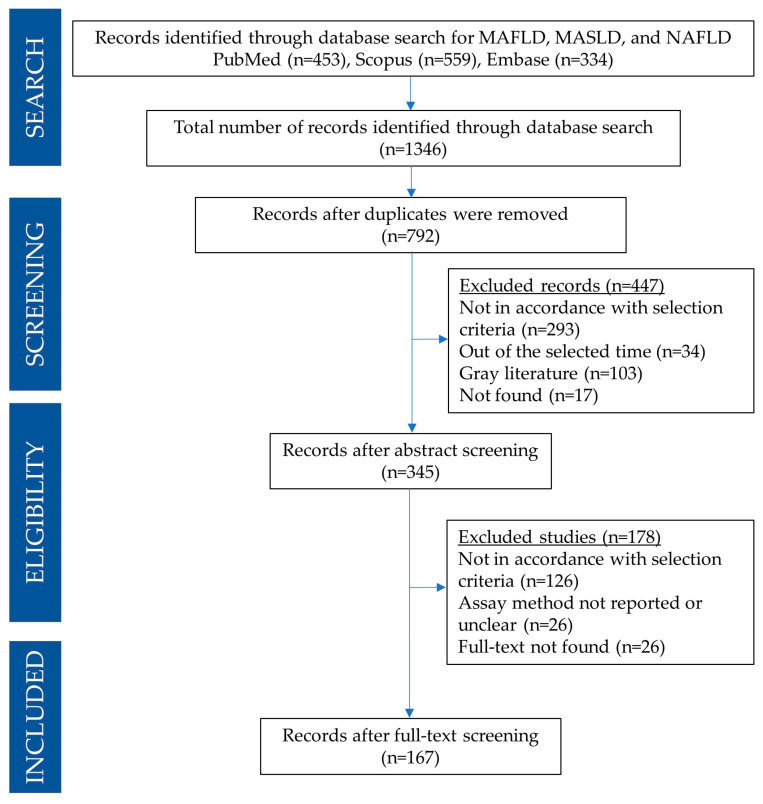
PRISMA flow diagram detailing the search and selection process applied during the overview.

A PRISMA flow diagram detailing the search and selection process for the present systematic review is reported in Figure 1. A total of 1346 articles were found in PubMed, Embase, and Scopus databases. In particular, 23 articles for MAFLD or MASLD query and 430 for NAFLD were found using the PubMed search engine; 24 articles for MAFLD or MASLD and 310 for NAFLD were found using the Embase search engine, and 45 articles for MAFLD or MASLD and 514 for NAFLD were found using Scopus’ search engine). Search results were imported into JabRef for further management [27].

After removing duplicates, the search amounted to 792 articles. Based on the title and abstract, 447 articles were excluded as they did not align with the focus of this review, following the previously outlined exclusion criteria. Subsequently, the remaining 345 articles were thoroughly manually examined. Based on the analysis of the full text, only 167 articles were found to be appropriate to be included in this systematic review.

### 3.2. Results Organization

All selected articles were initially categorised into three sections, depending on whether the study design included animal, cell models, or human fluids (tissue/plasma/serum/blood), and summarised in tables in chronological order. Next, the articles were divided into two macro areas depending on whether the molecule of interest was all forms of GSH or other LMW thiols (i.e., Cys, CysGly, and Hcy). Overall, Section 3.3, Section 3.4 and Section 3.5, as well as the included tables, described studies performed in animal models, cell models, and humans, respectively. Rats and mice studies that did not report explicit data and studies using other animals were included in Supplementary Tables.

### 3.3. Studies Evaluating GSH Levels in Animal Models of MASLD

Data extracted from articles studying all forms of GSH in animal models of MASLD were summarised in chronological order for rats in Table 1 [28,29,30,31,32,33,34,35,36,37,38,39,40,41,42,43,44,45,46,47,48,49,50,51,52,53,54] and Supplementary Table S1 [55,56,57,58,59,60,61,62,63,64,65,66,67,68,69,70,71,72,73,74,75,76,77,78,79,80,81,82,83,84,85,86,87,88,89,90,91,92,93,94,95,96,97,98,99,100,101,102] and for mice in Table 2 [103,104,105,106,107,108] and Supplementary Table S2 [109,110,111,112,113,114,115,116,117,118,119,120,121,122,123,124,125,126,127,128,129,130,131,132,133,134,135,136,137,138,139,140,141,142,143,144,145,146,147,148,149,150,151,152,153,154,155,156]. Additional studies using rabbits, fish, and primates were reported in Supplementary Table S3 [157,158,159,160]. In particular, each of the selected studies was firstly screened against the Critical Appraisal Skills Programme—CASP [161], and only when the article reached a score greater than or equal to 5 over ten questions were considered eligible for a detailed outline and main table.

#### 3.3.1. Studies on Rat Models

In this sub-section, we summarised the results of studies that evaluated GSH levels in MASLD models established in rats.

In a study by Khalaf et al. [28], the effects of allopurinol, metformin (MET), and vitamin E (VitE), both individually and in combinations, were investigated in a rat model of high-fructose diet (HFruD)-induced MASLD. All treatments, whether administered alone or in combination, restored the reduced hepatic GSH content in the model.

Palladini et al. [29] investigated changes in fatty acid desaturases, D5D, D6D, D9–16D, and D9–18D, and their relationship with oxidative stress on a rat model fed for 3 weeks with a methionine-choline-deficient (MCD) diet, evaluating hepatic oxidative stress parameters, such as reactive oxygen species (ROS) and GSH. In particular, GSH levels were reduced in the MCD group compared to control animals, and interestingly, GSH was positively correlated with D5 and D6.

Induction of hepatic oxidative stress and reduction of GSH levels were also confirmed in a MASLD rat model induced by a high cholesterol and fat-enriched diet (HCHF) [30]. Carvedilol and nicorandil counteracted these effects by lowering NF-kb expression and malondialdehyde (MDA) levels and increasing GSH content and endothelial Nitric oxide synthases (eNOS) expression, proving that vasodilatation could ameliorate MASLD.

Park et al. [31] evaluated the effects of mulberry water extracts (MB) combined with silk amino acids (SA) on Sprague Dawley rats fed with an HFD to induce MASLD. Measured reduced hepatic GSH was significantly lower in the HFD groups, but different ratios of treatments improved the liver damage and the GSH levels. Interestingly, MS1:3 exhibited a higher hepatic GSH content.

In a model where MASLD was achieved by administering 30% fructose in drinking water to rats, Pai et al. [32] demonstrated that the disease model exhibited a marked reduction in hepatic GSH levels with respect to the control group and that most of the hepato-metabolic effects were facilitated by the antioxidant and anti-inflammatory chrysin (chry).

Meng et al. [33] reported that treatment with *Cassia semens* prevented the histological damage and the reduction of the hepatic levels of GSH in rats with HFD-induced MASLD.

Souza Cruz et al. [34] investigated the long-term effects of ingestion of a 40% sucrose solution on serum and hepatic parameters in male Wistar rats. The study highlighted alteration in the oxidative stress markers, such as the decrease of all forms of GSH, alongside increased fibrotic tissue frequently described in MASLD.

Faheem et al. [35] induced MASLD in an HFD-fed rat model and observed significant hepatoprotective effects, as evidenced by the improved histopathological changes and restoration of oxidative stress markers (e.g., hepatic GSH) after treatment with cranberry.

The levels of LMW thiols were also evaluated in more complex models of MASLD, which combined ovariectomy with an HFHF diet. Ovariectomised (OVX) rats on an HFHF diet had significantly higher hepatic levels of MDA and lower GSH than OVX and the control group [36].

The ameliorative effect of ethanolic extract of garden cress seeds (EEGS) was explored in a rat model by Ibrahim et al. [37]. The study demonstrated that the administration of EEGS had hepatoprotective, antioxidant, and anti-steatosis characteristics in HFD-induced MASLD by restoring the hepatic GSH levels.

Ogunlana et al. [38] reported a significant reduction of GSH levels in the liver of HFD rats compared to the control rats. Still, different treatments, including pioglitazone (PIO), Ruzu herbal bitters (RUZU), and fenofibrate (FENO), counteracted this effect.

Also, MET alone or combined with phosphodiesterase inhibitors protected against hepatic-metabolic damage and restored hepatic GSH levels in rats with HFD-dependent MASLD [39].

The HFD-induced MASLD was also recovered by lycopene (lyc) supplementation. Indeed, Saeed et al. [40] demonstrated that the treatment with lyc hampered the lowering of GSH hepatic levels caused by HFD.

HFD has been linked to an imbalance in the intestinal microbiota, which may contribute to the pathogenesis of MASLD [41]. Moreover, the authors reported that restoration of eubiosis by supplementation with probiotic banana juice (PPBJ) significantly improved liver damage, reduced oxidative stress, and restored GSH to normal levels in the liver of rats fed with HFD.

The liver-brain axis was a further example of cross-talk among organs during MASLD pathogenesis. Jaleel et al. [42] explored the therapeutic effect of melatonin (MEL) on hepato- and neuro-complications in a rat model of MASLD induced by HFHF. The treatment with MEL improved several liver metabolites, neurotransmitters, and liver/brain GSH levels in the HFHF model.

In another model of fructose-induced MASLD, no significant differences in GSH were observed despite the reported hepato-metabolic effects [43].

In an HFD-fed MASLD rat model developed by Fawzy et al. [44], the treatment with eugenol successfully counteracted the histopathological lesions and the alterations of oxidative stress parameters (e.g., GSH hepatic levels).

Mengesha et al. [45] developed a MASLD model by administering a 20% fructose solution to Wistar rats. Owing to its hepatoprotective properties, this model restored dyslipidaemia and steatosis and altered hepatic GSH levels after silymarin (sily) treatment.

Coconut oil (CO) and thermally oxidized CO (TCO) integrated in an HFD and combined with streptozotocin (STZ) injection induced MASLD in rats [46]. Increased levels of hepatic GSH were found in the disease model groups, mainly when the liver tissue was extracted from a high-fat area.

Carvalho et al. [47] investigated how the concentration of fructose and the duration of exposure may influence the histological grading of hepatic microsteatosis and metabolic parameters in rats. Fructose consumption affected redox status, with GSH levels decreasing with increasing concentration and duration of exposure.

In an HFHF-induced MASLD rat model, Abd-Elrazek et al. [48] reported liver damage accompanied by a significant reduction in hepatic GSH levels compared to control rats, which were subsequently restored following treatment with sily, curcumin, and celery extracts.

Zakaria et al. [49] compared the protective and therapeutic effects of orlistat administration in an HFD-fed rat model. As expected, oxidative stress markers, including GSH, worsened after the diet. However, GSH levels were significantly increased in the treatment groups compared to the HFD group.

GSH levels in the hepatic tissues were also evaluated in a model of progressive MASLD induced by MCD in rats. GSH values at different time points were consistently lower than the corresponding controls. Interestingly, there was an inverse correlation between GSH and Fe levels but no correlation with Zn [50].

According to a previously mentioned study [32], two other studies evaluated the effects of chry on models of MASLD in rats. Attia et al. [51] induced MASLD by using HFruD, thus leading to a redox imbalance in the liver, evidenced by GSH depletion and the aggravation of other measured markers significantly improved by chry. Oriquat et al. [52], by obesogenic diet-induced MASLD, reported alterations in liver features and all forms of hepatic GSH, which were partially reverted after treatment with chry.

Reda et al. [53] induced MASLD using an HFD combined with fructose water, which exacerbated oxidative parameters, such as the reduction of hepatic GSH. Treatment with vitamin D improved these effects, thus reducing hepatic inflammation and steatosis.

Another study investigating the effect of natural extracts on HFD-induced MASLD in rats reported that *Matricaria pubescens* suppressed the hepatic damage and significantly increased GSH levels [54].

#### 3.3.2. Studies on Mice Models

Analogous to rats, mice models of MASLD may be established by using different dietetic regimens, and in the present paragraph, we reported studies that analyse GSH amounts.

Liu et al. [103] established a mice model fed with an HFHC to evaluate the protective effects of 14-deoxy-11,12-didehydroandrographolide (deAND) on MASLD-related liver injury. The disease model revealed increased oxidative stress and liver damage markers, and deAND treatment ameliorated these conditions, as evidenced by hepatic GSH patterns.

Several tea extracts were tested as treatments for the HFD-induced MASLD in a murine model. As expected, some of them may exert multiple actions on liver homeostasis, thus preventing liver impairment and diet-dependent reduction of GSH levels [104].

A daily HFD in combination with ethanol was used by Sukkasem et al. [105] to explore the potential therapeutic effects of hesperidin and myricetin against MASLD in mice. Notably, GSH levels and GSH/GSSG ratio were improved by all treatments.

Kang et al. [106] investigated the pharmacological effects of water chestnut extracts (WC) on a murine model of HFD-induced MASLD. A significant reduction in the content of hepatic GSH was reported in the disease group. However, MASLD and diabetes-related complications were significantly and dose-dependently normalised by oral administration of WC.

Mak et al. [107] reported the effects of the natural compound swietenine (SW) on a diabetic MASLD mouse model induced by HFD combined with STZ. The disease group exhibited altered liver antioxidant markers, notably decreased levels of serum GSH, which were reversed by oral administration of SW.

A further study evaluated the effects of 5-aminoimidazole-4-carboxamide-1-β-D-ribofuranoside, an inhibitor of 5’-adenosine monophosphate-activated protein kinase (AMPK) in a mice model of MASLD induced by HFHF [108]. As described above, this dietetic regimen caused hepato-metabolic dysregulation and lowered GSH plasma levels, which were counteracted by the AMPK inhibitor.

### 3.4. Studies Evaluating GSH Levels in Cell Models of MASLD

Limited articles study all forms of GSH in models in vitro of MASLD. Most reported primary hepatocytes or hepatocyte-like cell lines treated with fatty acids (e.g., oleic acid and palmitic acid, alone or in combination) to mimic the steatosis in MASLD. In these models, often the authors evaluated the effect of different compounds with antioxidant properties. All these studies were summarised in chronological order in Table 3. We extrapolated the data from 25 studies, which evaluated the effects of pro-steatotic treatments, investigated the effects of some treatments in cell models, and measured GSH and or GSSG [58,66,68,92,144,147,148,151,162,163,164,165,166,167,168,169,170,171,172,173,174,175,176,177,178]. However, only the study by Balkrishna et al. [172] explicitly provided the GSH concentrations. In particular, the study evaluated the efficacy of livogrit, a tri-herbal Ayurvedic medicine, as a potential hepatoprotective agent against MASH-related hepatocellular damage, using HepG2 spheroids and rat primary hepatocytes. Results showed that livogrit effectively prevented disease damage by reducing lipid accumulation, ROS production, aspartate transferase release, and nuclear factor kappa B activation while increasing lipolysis, GSH levels, and mitochondrial membrane potential.

### 3.5. Studies Evaluating Cys, CysGly, Hcy, and Total Thiols in In Vivo and In Vitro Models of MASLD

Only data from three articles measuring other hepatic LMW thiols, including CysGly, Cys, and Hcy, were extrapolated [110,179,180]. Notably, all studies were conducted in murine models of MASLD, but only one study reported a statistically significant difference between the disease model and the control (Table 4).

Deng et al. [110] used an omics approach to investigate the impact of PCB-126 on liver metabolites in healthy mice and those with MCD-dependent MASLD. The authors reported that LMW thiol levels were similar between the control and model but found significant alteration when the disease model was treated with PCB-126. Similarly, Luciano-Mateo et al. [179] showed that the hepatic levels of Hcy remained unaltered in the HFD model with respect to control mice, even though CCL2 deficiency may affect the amount of these metabolites.

The third study evaluated the total amount of LMW thiols in the livers of control mice compared to HFHF mice [180]. The study demonstrated a reduction in total thiol concentration in the model, which was restored by treatment with lupeol or MET. Interestingly, lupeol also downregulated the expression of androgen receptors and toll-like receptors 2 and 4 (TLR), thus leading to antioxidant and anti-inflammatory responses.

### 3.6. Studies Evaluating LMW Thiols in Humans

Our search results revealed that a systematic review and meta-analysis recently reviewed the Hcy levels in human MASLD [181]. Therefore, here, we focused on evaluating observational studies that assessed GSH in plasma samples (Table 5) or clinical trials measuring GSH or other thiols as secondary endpoints in clinical trials conducted on adult and paediatric patients with MASLD (Table 6).

#### 3.6.1. Observational Studies

In particular, Table 5 reports studies investigating the circulating levels of LMW thiols in five observational case-control studies in adults affected by MASLD compared to healthy subjects [182,183,184,185,186,187].

Świderska et al. [182] investigated redox abnormalities in MASLD, focusing on enzymatic and non-enzymatic antioxidants, redox homeostasis, and oxidative damage in 67 patients. Results indicated significantly elevated levels of Cu-Zn-superoxide dismutase (SOD), glutathione peroxidase (GPx), glutathione reductase (GR), total oxidant status (TOS), advanced glycation end products (AGE), MDA, and DNA/RNA oxidative damage in both MASLD groups compared to controls. Surprisingly, in this study, the levels of the reduced form of GSH were significantly higher in patients with early and advanced MASLD than in controls.

The relationship between systemic oxidative stress, indicated by protein-adjusted serum-free thiol levels, and MASLD was investigated in a large cohort of 5562 patients [183]. The disease was defined using the Fatty Liver Index (FLI) differentiating patients with FLI < 60 from patients with FLI ≥ 60. Results showed that serum-free thiol levels were significantly lower in FLI ≥ 60 than in FLI < 60 individuals. Stratified analyses revealed that the relationship between thiol levels and MASLD varied by gender, hypertension, and hypercholesterolemia, and, additionally, lower thiol levels were strongly linked to an increased risk of all-cause mortality.

Masarone et al. [184] aimed to determine if metabolomic profiles could distinguish between different stages of MASLD (simple steatosis, steatohepatitis, cirrhosis) and controls. Metabolomic signatures were analysed in 69 controls and 144 patients with MASLD. The authors demonstrated that the primary metabolic derangements in the MASLD group included essential and non-essential amino acids, GSH and xanthine, free fatty acids, and short-chain fatty acids and their intermediates. All their pathways are linkable with the known pathophysiologic mechanisms of disease onset and progression.

The cross-sectional study by Arya et al. [185] aimed at comparing oxidative stress markers and antioxidant enzyme activity in 60 patients with MASLD versus 25 healthy individuals, finding significantly higher levels of alanine aminotransferase, MDA, and nitric oxide metabolites in MASLD patients, along with lower total thiol levels and SOD activity compared to the controls. Molecular docking analysis suggested that MDA may deactivate SOD1 by interacting with its active site, indicating that impaired antioxidant defences, particularly through the deactivation of SOD1 by MDA, may play a critical role in the progression of MASLD.

In children with MASLD associated with severe obesity who underwent laparoscopic sleeve gastrectomy (LSG), Pastore et al. [186] hypothesized that an additional factor linked to one-carbon metabolism, that could be related to the recovery of metabolic derangement and histological damage after LSG, could be associated with increased levels of reduced GSH. Accordingly, they found a trend of increase in plasma levels of tGSH and Hcy that correlated with several parameters that ameliorated after LSG in children.

Garcia et al. [187] evaluated the levels of ROS, GSH, and antioxidant enzyme activities in peripheral blood mononuclear cells, and CD4+ and CD8+ T-lymphocytes from patients with MASLD and control healthy subjects. Cells from MASLD patients showed higher ROS levels, increased GPx activity, and lower levels of GR, SOD, and GSH compared to controls. Resistin stimulation further decreased GSH content in blood cells with a major effect on the MASLD group, thus highlighting the key role of resistin in the disruption of redox homeostasis in patients.

#### 3.6.2. Clinical Trials

As shown in Table 6, most of the studies that investigated LMW thiols in humans are clinical trials in which the authors assessed not only the improvement of steatosis but also the amelioration of different anthropometric and metabolic parameters and changes in the oxidative stress circulating biomarkers [95,96,97,98,99,100,101,102].

In a randomized, double-blind, placebo-controlled trial involving children with biopsy-proven MASLD, Nobili et al. [188] aimed to assess the anti-steatogenic effects of a 4-month treatment with VitE and hydroxytyrosol (HXT). In particular, 80 paediatric patients with MASLD were enrolled in two arms: the treatment group receiving HXT and VitE, and the placebo group receiving placebo. Results showed that the treatment with HXT and VitE greatly improved steatosis, insulin resistance, triglyceride levels, and oxidative stress parameters, including GSH and GSSG.

Another double-blind, placebo-controlled trial evaluated the effects of pinitol supplementation on liver fat content in adults with MASLD. Treatment with low or high doses of pinitol significantly reduced liver fat content and liver enzymes. However, the authors did not find a statistically significant change in GSH levels. Still, they found GPx, pyroglutamic acid, and glutamate significantly decreased after the treatment compared to the placebo group [189].

Maharshi et al. [190], in a pilot study, demonstrated that in adult patients with MASLD, lifestyle standard management (SMT) or SMT combined with *H. pylori*-eradication therapy (HPET) exhibited after 24 weeks a comparable effect in reducing hepatic steatosis and liver enzymes, even if interestingly only the HPET induced significant increase of serum GSH levels.

A randomized cross-over clinical trial performed on obese children with MASLD demonstrated that a calorie-restricted regimen alone or coupled with lycopene-rich tomato sauce improved steatosis and metabolism, though these effects were more profound in the tomato-supplemented group [191]. Moreover, the authors reported that only tomato supplementation resulted in glycolytic metabolic activation of T-cells and a marked increase in serum GSH.

The effects of VSL#3^®^ probiotic supplementation on cardiovascular risk and liver injury biomarkers in patients with MASLD were investigated by Chong et al. [192] in a randomized, double-blinded, placebo-controlled study. Endothelial function, oxidative stress, inflammation, insulin resistance, and liver injury markers were measured before and after the intervention. No significant changes were observed in the markers of cardiovascular risk, fibrosis indexes, and levels of GSH blood following VSL#3^®^ supplementation.

On the other hand, Yurtdas et al. [193] assessed the impact of the Mediterranean diet (MD) versus a conventional low-fat diet (LFD) in adolescents with obesity and MASLD. Both diets significantly reduced hepatic steatosis, serum transaminase levels, and insulin resistance, while improving inflammation and oxidative stress markers. Specifically, the difference in GSH blood levels between the two groups was significant at the 12-week follow-up, with higher levels in MD vs. LFD.

Tavakoli et al. [194] evaluated oxidative stress markers in MASLD patients diagnosed using abdominal ultrasound before and after treatment with pioglitazone. The results showed that at diagnosis, MASLD patients had significantly higher MDA and thiol levels compared to the control group. However, after three months of treatment with pioglitazone, MDA levels decreased, while thiol levels increased highlighting the role of pioglitazone in reducing these oxidative stress markers.

According to a previous study [193], Quetglas-Llabrés et al. [195] examined the impact of an MD intervention on antioxidant and inflammatory markers in patients with MASLD. Forty adult patients were divided based on their adherence to the MD, and after the intervention, both groups achieved an improved lipid profile characterized by decreases in total cholesterol and triglyceride levels. However, only participants who achieved higher adherence to the MD also exhibited decreased levels of glucose and liver enzymes, and increased GSH blood levels.

## 4. Discussion

The onset and progression of various pathologies are strongly linked to LMW thiol levels, and the liver is crucial in regulating the production and distribution of most of these molecules (i.e., GSH, Cys, CysGly, and Hcy) to various organs. Consequently, it is unsurprising that alterations in LMW thiols could indicate oxidative stress and play a key role in the pathogenesis of multiorgan diseases, such as MASLD. Several mechanisms have been proposed to explain changes in the levels of LMW thiols in MASLD [6]. GSH metabolism is regulated by the expression/activity of several enzymes (e.g., GSH peroxidase and GSH reductase), and it is susceptible to their gene expression by NRF2 transcription, whose knockout may reduce hepatocellular fat accumulation in experimental models [196]. The serum alteration of Cys and Hcy levels could be influenced directly by the GSH levels or other mechanisms, including the remethylation cycle and epigenetic control [186]. Even though the pathways acting as upstream regulators of LMW thiols may offer plausible therapeutic targets, they still remain poorly explored.

Hence, this systematic review was mainly focused on presenting a comprehensive summary of findings related to the assessment of LMW thiol levels in MASLD by analysing experimental studies conducted in in vivo and in vitro models and human subjects. A qualitative analysis of the included studies was also performed and reported in the next paragraph.

### 4.1. Qualitative Analysis

**Figure 2:**
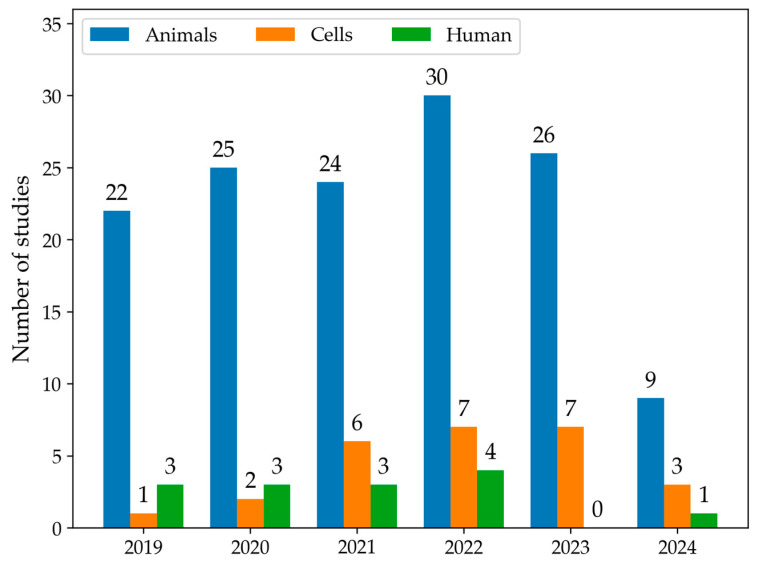
The histogram summarises the number of studies that measure LMW thiols using animal (blue) or cell models (orange) or conducted in human samples (green) from January 2019 to June 2024.

The first qualitative analysis examined the number of articles that discussed data on LMW thiols in MASLD from 2019 to 2024, categorising studies by models or human samples. As shown in Figure 2, the number of eligible articles increased each year. Moreover, animals were the most common experimental MASLD model employed in studies, followed by cellular lines. In 2019, a total of 26 articles were published. Among these articles, 22 used animal models, one used cell models, and three studies were conducted in humans. In 2022, there was an increase in the total number of articles to 41, including 30 articles in animal models, 7 in cell models, and four studies in human samples. Overall, animal models were the most commonly used across all years. The use of cell models showed a gradual increase, particularly from 2021 onward. Human studies remained low throughout the years. The number of articles selected for 2024 only accounts for the year’s first half; therefore, the number is smaller.

**Figure 3:**
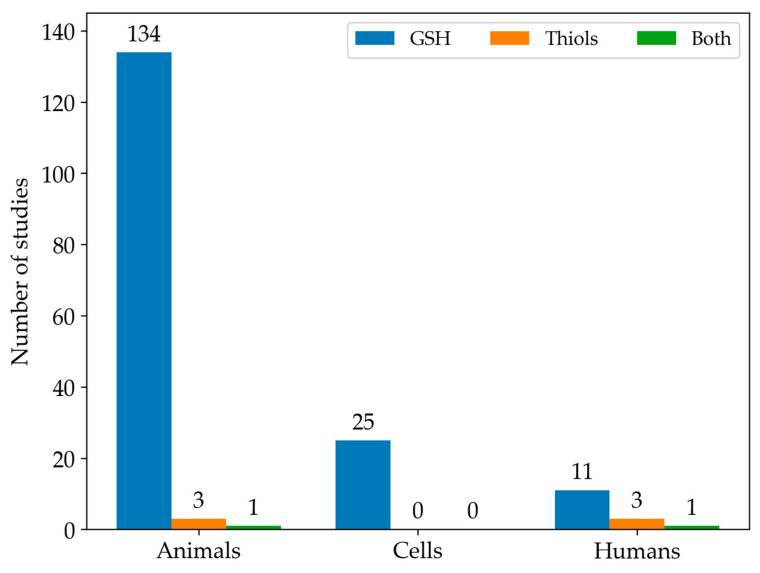
The histogram shows the distribution of studies focused on GSH levels (blue), other LMW thiol levels (orange), or both (green) in animal, cell, and human models.

Among the studies we found eligible for this systematic review, GSH was the most commonly analysed LMW thiol. In contrast, only 3% of the studies measured the full spectrum of LMW thiols. In particular, Figure 3 illustrates the number of articles that measured either GSH or all LMW thiols across different experimental types of samples.

**Figure 4:**
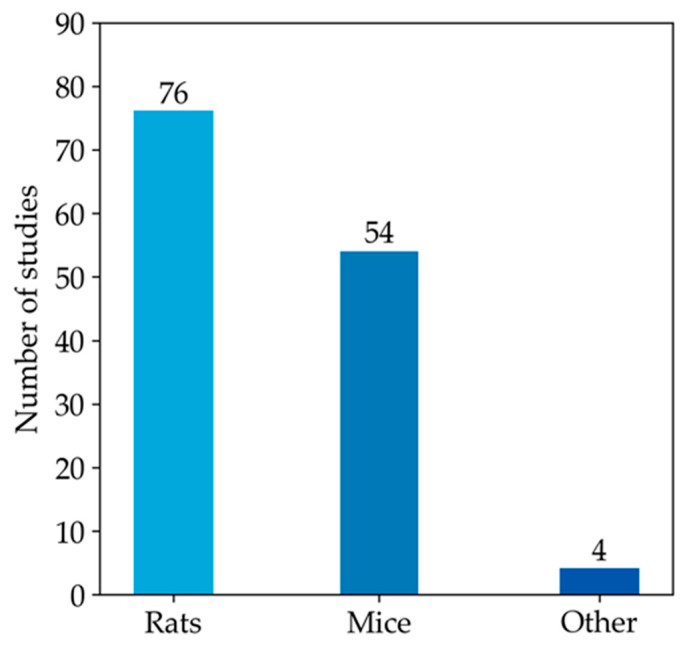
The histogram represents the number of articles measuring GSH levels in rat or mouse models. The remaining four articles in the ‘other’ category include studies that investigate GSH levels in monkey, rabbit, and zebrafish models.

**Figure 5:**
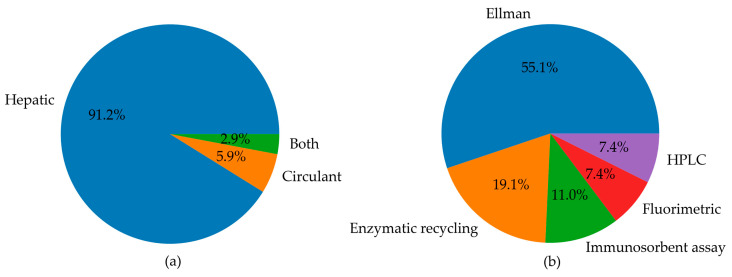
Pie charts summarising (**a**) the number of studies measuring circulating and hepatic levels of GSH, or both, in animal models; (**b**) the landscape of methods used for the determination of GSH levels in animal models, including Ellman’s method, enzymatic recycling, immunosorbent assays, fluorimetric analysis, and HPLC.

Since most studies focused on the measurement of GSH in animal models, the rest of our qualitative analysis was conducted only on these articles. Among animal studies (Figure 4), rats were the most commonly used animal model, specifically in 76 studies, followed by mice, featured in 54 articles. In contrast, other models (i.e., monkeys, rabbits, and zebrafish) were used much less frequently. As emerged from the evaluation of the animal studies, changes in GSH levels were different in direction and amount. These discrepancies depend on the fact that animal models of MASLD were established using different diets, mainly enriched in lipid or carbohydrate content, but at varied percentages. Moreover, other study design variables, such as the age of animals and length of study, can also influence the outcome of model induction and treatment, thus influencing the amount of GSH in both liver and blood samples [197]. Moreover, GSH was analysed using different types of sample matrices and various methods. Figure 5a reports the number of studies measuring GSH levels in blood samples (i.e., blood cells, plasma, serum) compared to the studies assessing GSH amounts in hepatic tissue. In particular, GSH is mainly measured in hepatic tissue (91.7%), whereas studies estimating the LMW thiol in serum, plasma, or blood are less frequent (5.3%). A few studies analysed GSH in both sample matrices (3.0%).

The distribution of various methods used for GSH level detection was reported in Figure 5b. The pie chart highlights that Ellman’s method was the most commonly used to analyse GSH levels (55.1%). The enzymatic recycling method and immunosorbent assays were also frequently used in 19.1% and 11% of studies, respectively. HPLC and fluorimetric techniques were employed collectively in 14.8% of studies. This latter analysis of methods used in the selected article reveals the pitfalls in the GSH analysis and emphasises the need for standardisation and reproducibility tools. The major weak points in GSH determination are the ease of non-enzymatic GSH autoxidation at pH > 7 and enzymatic conversion of GSH, the first step of which is mediated by GGTs, which exhibit optimal activity at neutral pH. Thus, it is essential to maintain the pH of the media in the acidic range [12]. The measurement of GSH and GSSG in biological samples [9,198] requires thus caution to prevent assay artefacts or data misinterpretation. Accordingly, the following points must be checked to achieve accurate analyses of GSH and GSSG: sample collection, reduction of disulphides, and, if needed, deproteinisation. Not all the literature cited points out this essential step in the methods section. Another critical point in GSH analysis is the method used. Below, we report the methods utilised in the literature cited in this review. Analytical methods that use colourimetric reagents and UV–VIS absorbance detection are inferior in sensitivity but simple compared with electrochemical or fluorometric determination. Ellman’s reagent (5,5′-dithio-(bis-2-nitrobenzoic) acid, DTNB) is widely used for the analysis of thiols in biological samples via the determination of the liberated anion [10].

Tietze [11] published his classical spectrophotometric method in 1969, often called the GR-coupled enzymatic recycling assay or the GSH-recycling assay. The GR-coupled recycling assay is still one of the most widely applied techniques to detect GSH and GSSG due to its simplicity, satisfactory sensitivity, and low cost. Several spectrofluorimetric methods have been developed to analyse GSH, GSSG, and related compounds in different matrices [12]. Several fluorophores were used, such as the dithiolic fluorophore and other rhodamine-based fluorescent probes [199], most reacting with all the cell’s thiol functionalities. The HPLC, coupled with a fluorometric, spectrophotometric, or mass spectrometry detector, has recently been the method of choice for measuring GSH and related thiols in biological samples. The HPLC techniques are rapid, highly specific, sensitive (0.5 pmol order), and reproducible. The simultaneous determination of GSH and other thiols in a single assay may be achieved by the appropriate choice of column, derivatisation, elution protocols, and detection system [12]. Finally, a few papers used an immunosorbent method for GSH assay. This method has not been validated against a gold standard but seems precise and sensitive. Anyway, it only allows reduced GSH determinations.

**Figure 6:**
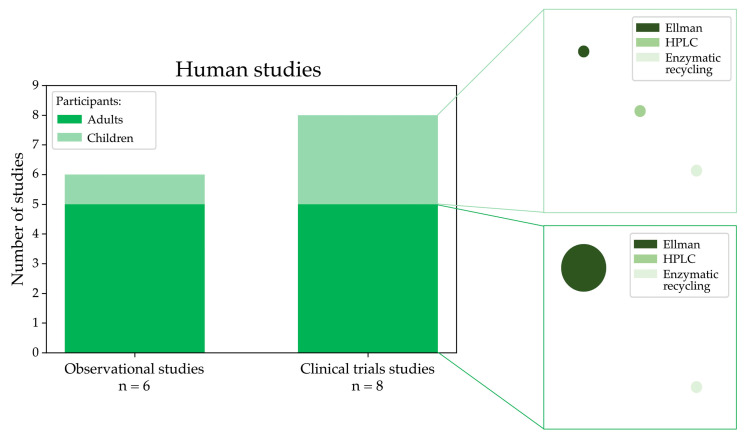
Graphical visualisation of the number of observational and clinical trials measuring LMW thiols conducted in humans affected by MASLD categorised into adult and paediatric groups and the experimental methods used for the analysis (the circle size is proportional to the number of studies using a specific methodology).

In this systematic review, we also selected 14 studies that analysed LMW thiols using samples of patients affected by MASLD and relative controls. Figure 6 shows the distribution of study types and participant demographics in human studies selected in this review, highlighting that clinical trials and observational studies often focus on adults, with fewer studies involving paediatric populations. As evidenced in the plot, many studies used Ellman’s method for the analysis.

### 4.2. Limitations of the Study

Potential biases could include the type and number of selected databases used for the search. Only three databases (i.e., PubMed, Embase, and Scopus) were used for this systematic review. However, these databases are recognised as the principal resources for literature recording [200]. Another limitation could be the choice of eligible studies that could have been strongly influenced by several factors, including the availability of complete explicit data, methods, or an inadequate model to resemble MASLD. Moreover, the grey literature exclusion may represent an additional limitation, even though its inclusion could have emphasised the missing data problem. A further limitation of the present study may be represented by manual screening. An automated or AI-based literature screening might enhance the consistency and efficiency of eligible studies in the future.

Finally, the significant heterogeneity and data insufficiency have hampered the performance of a meta-analysis and the ability to obtain a picture of GSH levels in MASLD.

## 5. Conclusions

LMW thiols, particularly GSH, play pathogenic roles in various diseases. Central to the production and systemic distribution of LMW thiols, the liver is susceptible to oxidative stress that could trigger liver damage, leading to MASLD. In this systematic literature review of recent studies assessing the levels of LMW thiols in MASLD models and human subjects, we highlight heterogeneity in sample processing and measurement of LMW thiol levels, which does not allow to perform a meta-analysis and hinder the establishment of cut-offs that could be used for MASLD diagnosis and stratification. The introduction of standardisation of measuring methods is an imperative step to move forward future studies that unveil the pathogenetic role of these molecules and their translatability into a diagnostic flowchart.

## Figures and Tables

**Table 1:** Studies assessing reduced, oxidized, and total glutathione (GSH, GSSG, tGSH) levels in MASLD rat models.

Strain	Diet and/or Supplements	Study Length (Weeks)	GSH and GSSG Levels in Model(s) (Mean ± Standard Deviation)	GSH in Normal Diet (ND) (Mean ± Standard Deviation)	Method	*p*-Values	Ref.
Not reported (nr)	ND, high-fructose diet (HFruD), HFruD + allopurinol (A), HFruD + metformin (MET), HFruD + vitamin E (VitE), HFruD + MET + VitE, HFruD + A + MET + VitE	4	Hepatic GSH (mg/g tissue): HFruD: 107 ± 1.70 HFruD + A: 119.3 ± 2.77 HFruD + MET: 128.9 ±1.44 HFruD + VitE: 122.9 ± 1.76 HFruD + MET + VitE: 122.5 ± 1.67 HFruD + A + MET + VitE: 129.8 ± 1.30	Hepatic GSH (mg/g tissue): 129.7 ± 1.14 mg/g tissue	Ellman	Lower in HFruD vs ND and vs. all groups of treatments (*p* < 0.05)	[28]
Wistar and Zucker	ND, methionine and choline-deficient (MCD) diet on Wistar	3	Hepatic tGSH (nmol/mg protein): MCD: 16.1 ± 0.9 Lean Zucker: 35.7 ± 2.0 Obese Zucker: 31.2 ± 2.7	Hepatic tGSH (nmol/mg protein): 37.5 ± 1.6	Enzymatic recycling	Lower in MCD vs. ND (*p* < 0.05)	[29]
Sprague–Dawley	ND, cholesterol and fat-enriched (HCHF) diet, HCHF + carvedilol (CARV), HCHF + nicorandil (NICO)	4 (HCHF) + 4 (ND + No drug/HCHF + CARV/NICO)	Hepatic GSH (mmol/mg protein): HCHF: 28.38 + 2.120 HCHF + CARV: 43.08 + 5.301 HCHF + NICO: 33.13 + 2.446	Hepatic GSH (mmol/mg protein): 52.67 + 0.835	Ellman	Lower in HCHF vs. ND and vs. HCHF + CARV and HCHF + NICO (*p* < 0.05)	[30]
Sprague–Dawley	ND, high-fat diet (HFD), HFD + mulberry extract (MB), and silk amino acids (SA) mixtures	12	Hepatic GSH (μmol/g protein): HFD: 22.8 ± 2.1 HFD + MB/SA 1:3 low dosage: 25.1 ± 2.2 HFD + MB/SA 1:3 high dosage: 27.7 ± 2.1 HFD + MB/SA 1:5 low dosage: 23.5 ± 2.5 HFD + MB/SA 1:5 high dosage: 24.8 ± 2.2	Hepatic GSH (μmol/g protein): 24.9 ± 2.3	Ellman	- Lower in HFD vs. ND (*p* < 0.05) - Recovery with all treatments (mainly with SA 1:3) (*p* < 0.05)	[31]
Wistar	ND, HFruD, HFruD + chrysin (chry)	16	Hepatic GSH (μg/mg tissue protein): HFruD: 363.7 ± 71.67 HFruD + chry (25 mg/kg; 50 mg/kg; 100 mg/kg): 553.9 ± 32.35; 714.2 ± 39.75; 844.2 ± 89.6	Hepatic GSH (μg/mg tissue protein): 901 ± 97.45	Ellman	- Lower in HFD vs. ND (*p* < 0.001) - Recovery with treatments at higher doses (*p* < 0.05, *p* < 0.001)	[32]
Wistar	ND, HFD, HFD + MET, HFD + *Cassia semens* (Cs)	12	Hepatic GSH (mg/g protein): HFD: 4.55 ± 0.91 HFD + MET: 6.68 ± 1.26 Cs (0.5 g/kg; 1 g/kg; 2 g/kg): 5.88 ± 1.06; 6.79 ± 0.93; 7.79 ± 1.48	Hepatic GSH (mg/g protein): 9.76 ± 1.32	Ellman	- Lower in HFD vs. ND - Dose-dependent recovery with all treatments (*p* < 0.05, *p* < 0.01)	[33]
Wistar	ND, sucrose (S)	approximately 26	Hepatic GSH (nmol/mg tissue): S: 7.05 ± 0.76 Hepatic GSSG (nmol/mg tissue): S: 1.28 ± 0.06 Hepatic GSH + 2× GSSG (nmol GSH units/mg tissue): S: 8.33 ± 0.75 Hepatic GSH/GSSG: S: 5.57 ± 0.67	Hepatic GS (nmol/mg tissue): 18.54 ± 0.41 Hepatic GSSG (nmol/mg tissue): 2.38 ± 0.12 Hepatic GSH + 2× GSSG (nmol GSH units/mg tissue): 20.92 ± 0.50 Hepatic GSH/GSSG: 7.83 ± 0.28	Fluorimetric	Lower GSH, GSSG and GSH + 2× GSSG in sucrose vs. ND (*p* < 0.05)	[34]
Wistar	ND, high-fat cholesterol diet (HFCD), ND + cranberry (cra), HFCD + cra	8	Hepatic GSH (pg/mg protein): HFCD: 9.66 ± 0.60 ND + cra (100 mg/kg): 69.8 ± 3.34 protein HFCD + cra (50 mg/kg; 100 mg/kg): 19.01 ± 1.33; 34.47 ± 0.74	Hepatic GSH (pg/mg protein): 43.22 ± 1.42	Ellman	- Lower in HFD vs. ND - Dose-dependent recovery with all treatments (*p* < 0.05)	[35]
Sprague-Dawley	ND, ovariectomized (OVX) + ND, OVX + high-fat and high-fructose diet (OVX + HFHF)	4	Hepatic GSH (nmol/mg protein): OVX + ND: 55.21 ± 1.40 OVX + HFHF: 46.01 ± 0.91	Hepatic GSH (nmol/mg protein): 57.94 ± 0.32	Ellman	- Lower in OVX + ND vs. ND (*p* < 0.01) - Lower in OVX + HFHF vs. OVX + ND (*p* < 0.01)	[36]
nr	ND, HFD, ND + ethanolic extract of garden cress seeds, HFD + Garden Cress (GC)	12	Hepatic GSH (pg/g tissue) HFD: 29.64 ± 0.91 GC: 42.59 ± 1.64 HFD + GC: 38.18 ± 1.77	Hepatic GSH (pg/g tissue) 41.58 ± 2.48	Immunosorbent	Hepatic GSH: Lower in HFD vs. ND and vs. treatment group (*p* < 0.05)	[37]
Wistar	ND, HFD, HFD + pioglitazone (PIO), HFD + Ruzu herbal bitters (RUZU), HFD + fenofibrate (FENO)	12	Hepatic GSH (nmol/mg protein): HFD: 72.24 ± 5.15 HFD + PIO: 112.10 ± 3.79 HFD + RUZU: 119.19 ± 9.21 HFD + FENO: 151.53 ± 19.69	Hepatic GSH (nmol/mg protein): 112.18 ± 6.18	Enzymatic recycling	- Lower in HFD vs. ND (*p* < 0.05) - Recovery with all treatments (*p* < 0.05)	[38]
Wistar	HFD, HFD + MET, HFD + pentoxifylline (PTX) + MET, HFD + cilostazol (CLS) + MET, HFD + sildenafil (SLD) + MET	16	Hepatic GSH (mmol/g tissue): HFD + MET: 45.9 ± 2.0 HFD + PTX + MET: 69.9 ± 2.8 HFD + CLS + MET: 42.3 ± 5.0 HFD + SLD + MET: 50.5 ± 3.18	Hepatic GSH (mmol/g tissue): 74.4 ± 3.8	Ellman	- Lower in HFD vs. ND (*p* < 0.05) - Recovery with all treatments (*p* < 0.05)	[39]
Sprague-Dawley	ND, HFD, ND + lycopene (lyc), HFD + lyc	8	Hepatic GSH (pg/mg protein): HFD: 2.5 ± 0.35 ND + lyc: 13.0 ± 1.7 HFD + lyc: 8.3 ± 0.7	Hepatic GSH (pg/mg protein): 12.4 ± 1.2	Ellman	- Lower in HFD vs. ND (*p* < 0.05) - Recovery with treatment (*p* < 0.05)	[40]
Wistar	ND, HFD, ND + pectinase treated probiotic banana juice (PPBJ), HFD + PPBJ	20	Hepatic GSH (µmol/µg protein): HFD: 0.4 ± 0.21 ND + PPBJ: nr HFD + PPBJ: 1.04 ± 0.04	nr	Ellman	Lower in HFD vs. treatment control (*p* < 0.001)	[41]
Wistar	ND, HFHF, HFHF + MET, HFHF + melatonin (MEL)	8	Hepatic GSH (μmol/g tissue): HFHF: 78.32 ± 2.28 HFHF + MET: 99.31 ± 4.68 HFHF + MEL: 104.9 ± 4.24	Hepatic GSH (μmol/g tissue): 122.2 ± 6.58	High-performance liquid chromatography (HPLC)-UV	- Lower in HFHF vs. ND (*p* < 0.05) - Recovery with treatment groups (*p* < 0.05)	[42]
Wistar	ND, HFruD, ethanol, HFruD + 1,25-dihydroxyvitamin D3 (1,25(OH)2D3), ethanol + 1,25(OH)2D3	8	Hepatic GSH (nmol/mg protein): HFruD: 19.4 ± 4.03 ethanol: 21.5 ± 5.17 HFruD + 1,25(OH)_2_D_3_: 19.5 ± 4.99 ethanol + 1,25(OH)_2_D_3_: 26.9 ± 5.70	Hepatic GSH (nmol/mg protein): 23.9 ± 3.02	Ellman	Not significant	[43]
Wistar	ND, HFD, ND + eugenol (EUG), HFD + EUG	8	Hepatic GSH (pg/mg protein): HFD: 11.1 ± 1.33 ND + EUG: 48.5 ± 3.24 HFD + EUG: 27.0 ± 2.03	Hepatic GSH (pg/mg protein): 40.0 ± 5.69 pg/mg protein	Immunosorbent	- Lower in HFD vs. ND (*p* < 0.05) - Recovery with treatment (*p* < 0.05)	[44]
Wistar	ND, HFruD, ND + silymarin (sily), HFruD + sily	8	Hepatic GSH (μmol/g tissue): HFruD: 33.93 ± 0.91 ND + Sily (400 mg/kg): 36.53 ± 0.89 HFruD + sily (200 mg/kg; 400 mg/kg): 35.49 ± 0.98; 36.35 ± 0.93	Hepatic GSH (μmol/g tissue): 37.98 ± 1.07	Ellman	- Lower in HFruD vs. ND (*p* < 0.001) - Recovery with treatment groups (*p* < 0.05, *p* < 0.01)	[45]
Wistar	ND, HFD/coconut oil (CO) + streptozotocin (STZ), HFD/thermally oxidized CO (TCO) + STZ	4 (ND/HFD) + 4 (ND + No drug/HFD + STZ)	Hepatic GSH in 2 different areas (µmol/mg protein): HFD/CO + STZ: 10.85 ± 1.88; 7.15 ± 1.04 HFD/TCO + STZ: 9.53 ± 0.67; 5.52 ± 1.86	Hepatic GSH (µmol/mg protein): 4.73 ± 0.66 µmol/mg protein	Ellman	Higher in all groups vs. ND (*p* < 0.01)	[46]
Wistar	ND, HFruD	8 12	Hepatic GSH—8 weeks (µM/100 mg tissue): HFruD (10%; 30%; 60%): 51.58 ± 13.90; 56.61 ± 24.79; 83.95 ± 17.95 Hepatic GSH—12 weeks (µM/100 mg tissue): HFruD (10%; 30%; 60%): 98.33 ± 19.80; 86.11 ± 42.20; 177.67 ± 60.30	Hepatic GSH (µM/100 mg tissue): 66.95 ± 4.83 (8 weeks) 126.58 ± 18.13 (12 weeks)	Enzymatic recycling	Decreased GSH with concentration and time (*p* < 0.0001)	[47]
Sprague-Dawley	ND, HFHF, HFHF + sily, HFHF + celery, HFHF + curcumin (cur)	16 (ND/HFHF) + 4 (ND/HFHF + No drug/HFHF + sily/HFHF/HFHF + cur)	Hepatic GSH (μmol/g tissue): HFHF: 14.47 ± 0.1 HFHF + sily: 18.44 ± 0.09 HFHF + celery: 21.22 ± 0.12 HFHF + cur: 19.71 ± 0.08	Hepatic GSH (μmol/g tissue): ND: 25.7 ± 0.24	HPLC-UV	- Lower in HFHF vs. ND (*p* < 0.05) - Recovery in treatment groups (*p* < 0.05)	[48]
Sprague-Dawley	ND, HFD, HFD + orlistat (O), obese (ob)/HFD + O	6 (ob/HFD + O), 12 (HFD + O)	Hepatic GSH (nmol/mg protein): HFD: 2.37 ± 0.10 HFD + O: 3.06 ± 0.06 ob/HFD + O: 2.91 ± 0.19	Hepatic GSH (nmol/mg protein): 3.29 ± 0.08	Ellman	- Lower in HFD vs. ND (*p* < 0.05) - Recovery in protective and therapeutic treatment groups (*p* < 0.05)	[49]
Wistar	ND, MCD	2, 4, 8	Hepatic GSH (nmol/mg protein): MCD 2 wk: 19.33 ± 1.34 MCD 4 wk: 16.18 ± 1.15 MCD 8 wk: 17.99 ± 1.74	Hepatic GSH (nmol/mg protein): 2 wk: 37.31 ± 1.49 4 wk: 37.66 ± 2.53 8 wk: 36.92 ± 2.51	Enzymatic recycling	Lower in MCD vs. ND (*p* < 0.05)	[50]
Wistar	ND, HFruD, HFruD + chry	8	Hepatic GSH (mg/g tissue): HFruD: 9.6 ± 0.64 HFruD + chry (25 mg/kg; 50 mg/kg): 12.18 ± 0.35; 13.06 ± 0.24	Hepatic GSH (mg/g tissue): 13.9 ± 0.34	Ellman	- Lower in HFruD vs. ND (*p* < 0.001) - Recovery in protective and therapeutic treatment groups (*p* < 0.001)	[51]
Wistar	ND, obesogenic diet (ObD), ObD + chry	12 (ND/ObD) + 4 (ND + No drug/ObD/ObD + chry)	Hepatic tGSH (nmol/mg protein): ObD: 21.4 ± 1.8 ObD + chry (25 mg/kg; 50 mg/kg; 75 mg/kg): 23.7 ± 1.9; 24.7 ± 1.9; 25.0 ± 1.7 Hepatic GSH (nmol/mg protein): ObD: 17.5 ± 1.5 Ob/D + chry (25 mg/kg; 50 mg/kg; 75 mg/kg): 20.8 ± 1.7; 22.3 ± 1.8; 22.7 ± 1.6 Hepatic GSSG: ObD: 2.0 ± 0.2 nmol/mg protein; ObD + chry (25 mg/kg; 50 mg/kg; 75 mg/kg): 1.5 ± 0.2; 1.2 ± 0.2; 1.2 ± 0.1 Hepatic GSH/GSSG: ObD: 8.9 ± 0.6 ObD + chry (25 mg/kg; 50 mg/kg; 75 mg/kg): 14.1 ± 1.2; 19.0 ± 2.5; 19.7 ± 1.6	Hepatic tGSH (nmol/mg protein): 27.7 ± 1.7 Hepatic GSH: 25.6 ± 1.6 Hepatic GSSG: 1.1 ± 0.1 Hepatic GSH/GSSG: 23.6 ± 2.0	Enzymatic recycling	tGSH and GSH/GSSG: - Lower in ObD vs. ND (*p* < 0.05) - Recovery in all treatments (*p* < 0.05) GSH: - Higher in ObD vs. ND (*p* < 0.05) - Recovery in all treatments (*p* < 0.05)	[52]
nr	ND, HFHF, vitamin D (VitD), HFHF + VitD	10	Hepatic GSH (mg/g.tissue): HFHF: 3.30 ± 0.20 VitD: 5.57 ± 0.15 HFHF + VitD: 4.61 ± 0.16	Hepatic GSH (mg/g tissue): 5.56 ± 0.29 mg/g tissue	Ellman	Lower in HFD vs. ND and treatment control (*p* < 0.05)	[53]
Wistar	ND, HFD, ND + *M. pubescens* powder (MP), HFD + MP	16	Hepatic GSH (nmol/mg protein): HFD: 48.95 ± 3.64 ND + MP: 78.68 ± 2.31 HFD + MP: 76.19 ± 4.44	Hepatic GSH (nmol/mg protein): 82.07 ± 4.09	Ellman	- Lower in HFD vs. ND (*p* < 0.001) - Recovery in the treatment group (*p* < 0.01)	[54]

GSH: reduced glutathione; GSSG: oxidized glutathione; ND: normal diet; HFruD: high-fructose diet; A: allopurinol; MET: metformin; VitE: vitamin E; MCD: methionine-choline–deficient; tGSH: total glutathione; HCHF: cholesterol and fat-enriched diet; CARV: carvedilol; NICO: nicorandil; HFD: high-fat diet; MB: mulberry extract; SA: silk amino acids; chry: chrysin; Cs: *Cassia semens*; S: sucrose; HFCD: high-fat cholesterol diet; cra: cranberry; OVX: ovariectomized; HFHF: high-fat and high-fructose diet; nr: not reported; GC: *Garden Cress*; PIO: pioglitazone; RUZU: Ruzu herbal bitters; FENO: fenofibrate; PTX: pentoxifylline; CLS: cilostazol; SLD: sildenafil; lyc: lycopene; PPBJ: pectinase treated probiotic banana juice; MEL: melatonin; HPLC: high-performance liquid chromatography; 1,25(OH)2D3: 1,25-dihydroxyvitamin D3; EUG: eugenol; sily: silymarin; CO: Coconut oil; STZ: streptozotocin; TCO: thermally oxidized; O: orlistat; ob: obese; ObD: obesogenic diet; VitD: vitamin D; MP: *M. pubescens* powder.

**Table 2:** Studies assessing the levels of GSH forms in mice models of MASLD.

Strain	Diet and/or Supplements	Study Length (Weeks)	GSH and GSSG Levels in Model(s) (Mean ± Standard Deviation)	GSH in Normal Diet (ND) (Mean ± Standard Deviation)	Method	*p*-Values	Ref.
C57BL/6J	ND, high-fat and high-fructose diet (HFHF), HFHF + 14-deoxy-11,12-didehydroandrographolide (deAND)	7	Hepatic GSH (nmol/mg protein): HFHC: 0.26 ± 0.03 HFHC + 0.05% deAND: 0.24 ± 0.05 HFHC + 0.1% deAND: 0.32 ± 0.04	Hepatic GSH (nmol/mg protein): 2.81 ± 0.99	High-performance liquid chromatography-mass spectrometry (HPLC-MS)	Lower in HFHF vs. ND, and vs. HFHF + deAnd at the higher dose (*p* < 0.05)	[103]
C57BL/6J	High-fat diet (HFD) + Gongmei White Tea (T1), White Peony Tea (T2), Enshi Yulu Tea (T3), Fried Green Tea (T4), Yihong Tea (T5), Lapsang Souchong Tea (T6), Wuyi Narcissus Tea (T7), Fenghuang Narcissus Tea (T8), Qing Brick Tea (T9), Pu-erh Tea (T10), Yuan’an Luyuan Tea (T11), Mengding Huangya Tea (T12)	15	Hepatic GSH (μmol/g protein): HFD: 3.94 ± 1.14 HFD + T1: 3.46 ± 0.79 HFD + T2: 3.76 ± 0.92 HFD + T3: 3.82 ± 0.40 HFD + T4: 2.58 ± 0.65 HFD + T5: 2.16 ± 0.37 HFD + T6: 4.63 ± 1.90 HFD + T7: 4.91 ± 1.62 HFD + T8 5.30 ± 0.98 HFD + T9: 3.80 ± 1.07 HFD + T10: 4.23 ± 0.76 HFD + T11: 4.53 ± 1.36 HFD + T12: 4.37 ± 1.38	Hepatic GSH (μmol/g protein): 5.26 ± 2.10	Ellman	Lower in the HFD group vs. ND and ND, and vs. T4, T5, and T8 (*p* < 0.05)	[104]
ICR	ND, HFD/ethanol, HFD/ethanol + fenofibrate (F), HFD/ethanol + hesperidin (H), HFD/ethanol + myricetin (M)	Approximately 8	Hepatic tGSH (nmol/mg protein): HFD/ethanol: 133.91 ± 23.82 HFD/ethanol + F: 92.69 ± 11.56 HFD/ethanol + H (50 mg/kg; 200 mg/kg): 98.83 ± 24.95; 73.71 ± 16.56 HFD/ethanol + M (50 mg/kg; 200 mg/kg): 83.52 ± 11.58; 82.43 ± 17.53 Hepatic GSH (nmol/mg protein): HFD/ethanol: 41.42 ± 9.20 HFD/ethanol + F: 63.58 ± 3.87 HFD/ethanol + H (50 mg/kg; 200 mg/kg): 69.03 ± 9.10; 35.63 ± 4.51 HFD/ethanol + M (50 mg/kg; 200 mg/kg): 48.07 ± 9.19; 48.87 ± 7.35 Hepatic GSSG (nmol/mg protein): HFD/ethanol: 42.11 ± 11.22 HFD/ethanol: + F: 29.11 ± 8.67 HFD/ethanol + H (50 mg/kg; 200 mg/kg): 29.80 ± 8.67; 38.08 ± 6.82 HFD/ethanol + M (50 mg/kg; 200 mg/kg): 35.45 ± 5.76; 33.56 ± 6.18 Hepatic GSH/GSSG: HFD/ethanol: 0.98 ± 0.11 HFD/ethanol: + F: 2.18 ± 0.57 HFD/ethanol + H (50 mg/kg; 200 mg/kg): 2.32 ± 0.31; 0.94 ± 0.48 HFD/ethanol + M (50 mg/kg; 200 mg/kg): 1.35 ± 0.44; 1.45 ± 0.53	Hepatic tGSH (nmol/mg protein) 133.91 ± 13.74 Hepatic GSH (nmol/mg protein) 104.86 ± 13.01 Hepatic GSSG (nmol/mg protein) 29.05 ± 5.00 Hepatic GSH/GSSG 3.61 ± 1.16	Enzymatic recycling	GSH, GSH/GSSG: Lower in HFD/ethanol vs. ND and vs. low dose H and vs. F (*p* < 0.05)	[105]
CD1 (ICR)	ND, HFD, HFD + metformin (MET), HFD + water chestnut (WC)	13	Hepatic GSH (µM/mg tissue): HFD: 10.45 ± 1.80 HFD + MET: 32.31 ± 10.11 HFD + WC (50 mg/kg, 100 mg/kg, 200 mg/kg): 10.74 ± 3.21; 32.31 ± 13.52; 45.49 ± 13.68	Hepatic GSH (µM/mg tissue): 69.63 ± 10.20	Ellman	Lowers in HFD vs. ND and vs. treatments at higher doses	[106]
C57BL/6J	ND, HFD/streptozotocin (STZ), HFD/STZ + swietenine (SW)	3 (HFD/STZ) + 8 (HFD + STZ/HFD + STZ + SW)	Serum GSH (nmol/µL): HFD/STZ: 0.71 ± 0.18 HFD/STZ + SW: 2.02 ± 0.08	Serum GSH (nmol/µL): 2.41 ± 0.21	Ellman	Lower in HFD/STZ vs. ND and vs. treatment (*p* < 0.0001)	[107]
C57BL/6	ND, ND + 5-aminoimidazole-4-carboxamide-1-β-D-ribofuranoside (AICAR) HFHF, HFHF + AICAR	10	Plasma GSH (μg/mg protein): AICAR: 9.80 ± 0.22 HFHF: 5.72 ± 0.17 HFHF + AICAR: 7.63 ± 0.19	Plasma GSH (μg/mg protein): 9.78 ± 0.20	Ellman	Lower in HFHF vs. ND and vs. treatment (*p* < 0.05)	[108]

GSH: glutathione; GSSG: oxidized glutathione; ND: normal diet; HFHF: high-fat and high-fructose diet; deAND: 14-deoxy-11,12-didehydroandrographolide; HPLC-MS: high-performance liquid chromatography-mass spectrometry; HFD: high-fat diet; T1: Gongmei White Tea; T2: White Peony Tea; T3: Enshi Yulu Tea; T4: Fried Green Tea; T5: Yihong Tea; T6: Lapsang Souchong Tea; T7: Wuyi Narcissus Tea; T8: Fenghuang Narcissus Tea; T9: Qing Brick Tea; T10: Pu-erh Tea; T11: Yuan’an Luyuan Tea; T12: Mengding Huangya Tea; F: fenofibrate; H: hesperidin; M: myricetin; MET: metformin; WC: water chestnut; STZ: Streptozotocin; SW: swietenine; AICAR: 5-aminoimidazole-4-carboxamide-1-β-D-ribofuranoside.

**Table 3:** Cell studies on GSH involvement in MASLD.

Model	Supplements	Study Length (Hours)	GSH and GSSG Levels in Model(s)	GSH in Control Group (Ctrl)	Method	*p*-Values	Ref.
BRL-3A cells	Ctrl, nonesterified fatty acids (NEFAs)	12	Not reported (nr)	nr	Ellman	GSH: Lower in NEFAs vs. Ctrl (*p* < 0.05)	[58]
HepG2 cells	Oleic acid (OA), OA + berbamine, OA + fenofibrate (FENO)	48	nr	nr	Fluorimetric	GSH: - Lower in OA vs. Ctrl (*p* < 0.05) - Recovery with treatments at higher dose (*p* < 0.05)	[162]
HepG2 cells	OA/tert-butylhydroperoxide (t-BHP), t-BHP + water extract of *A. annua*	6	nr	nr	Ellman	*GSH:* Lower in t-BHP vs. treatment groups (*p* < 0.001)	[163]
HepG2 cells	Ctrl, palmitic acid (PA), phloroglucinol (PHG), PA + PHG, PA + α-lipoic acid (ALA), PA + N-acetylcysteine (NAC) + H_2_O_2_	16	nr	nr	Enzymatic recycling	tGSH: Lower in PHG vs. Ctrl (*p* < 0.05) GSH: - Lower in PA (*p* < 0.0001) and PHG (*p* < 0.01) - Recovery dose-dependently with treatments (*p* < 0.01) GSSG: - Higher in PA vs. Ctrl - Recovery dose-dependently with treatments (*p* < 0.01) GSH/GSSG: - Lower in PA and PHG vs. Ctrl - Recovery dose-dependently with treatments (*p* < 0.05)	[164]
L02 cells	Ctrl, H_2_O_2_, H_2_O_2_ + *Lycii fructus* polysaccharide (LFP)	48	nr	nr	Ellman	GSH: - Lower in H_2_O_2_ vs. Ctrl (*p* < 0.0001) - Recovery with all treatments (*p* < 0.01, *p* < 0.001)	[165]
L02 cells	Free fatty acids (FFAs), FFAs + malvidin-3-O-glucoside (M3G), FFAs + malvidin-3-O-galactoside (M3Ga), FFAs + buthionine sulfoximine (BSO), FFAs + NAC	48	nr	nr	Fluorimetric	GSH: - Lower in FFAs and FFAs + BSO vs. Ctrl (*p* < 0.05) - Recovery with M3G, M3Ga and NAC (*p* < 0.05)	[166]
HepG2 cells	Ctrl, OA, OA + hesperetin (H)	24	nr	nr	Ellman	GSH: Lower in OA vs. Ctrl and vs. OA + H (*p* < 0.05)	[66]
HepG2 cells	FFAs, FFAs + picroside II (PIC), FFAs + silibinin	22	nr	nr	Enzymatic recycling	tGSH: - Lower in FFAs vs. Ctrl (*p* < 0.01) - Recovery with treatments (*p* < 0.05) GSH/GSSG: - Lower in FFAs vs. Ctrl (ns) and vs. FFAs + PIC (*p* < 0.01)	[167]
L02 cells	Ctrl, 5% fat emulsion (FE), FE + Jiuzhuan Huangjing pills	24	nr	nr	Ellman	GSH: - Lower in FE vs. Ctrl (*p* < 0.05) - Recovery with treatment at higher doses (*p* < 0.01)	[68]
Primary mouse hepatocytes	Ctrl, fructose (Fru), Fru + carminic acid (CA)	nr	nr	nr	Ellman	GSH: Lower in Fru vs. Ctrl and Fru + CA groups (*p* < 0.05)	[168]
HepG2 cells	Ctrl, FFAs, FFAs + xiaoheiyao (XHY-1)	24	nr	nr	Enzymatic recycling	GSH/GSSG: - Lower in FFAs vs. Ctrl - Recovery with treatment at higher doses (0.05)	[169]
HepG2 cells subcultured on rat liver biological matrix scaffolds	Ctrl, FFAs, FFAs + baicalin	192	nr	nr	Ellman	GSH: - Lower in FFAs vs. Ctrl (*p* < 0.05) - Recovery with treatment (*p* < 0.01)	[170]
L02 cells	Ctrl, PA	nr	nr	nr	Enzymatic recycling	GSH/GSSG: Lower in PA vs. Ctrl (significance nr)	[171]
HepG2 spheroids	Ctrl, methionine and cystine deficient media (MCDM), MCDM + livogrit, MCDM + pioglitazone	72	GSH, mean ± standard deviation (µmol/L): MCDM: 3.05 ± 0.14 MCDM + livogrit: 4.82 ± 0.13 MCDM + pioglitazone: 3.73 ± 0.01	GSH, mean ± standard deviation (µmol/L): 7.83 ± 1.26	Fluorimetric	GSH: - Lower in MCDM vs. Ctrl (*p* < 0.01) -Recovery with livogrit at higher doses (*p* < 0.001) and pioglitazone (*p* < 0.05)	[172]
L02 cells	Ctrl, cholesterol (CHO)	24	nr	nr	Enzymatic recycling	GSSG/GSH Higher in CHO vs. respective Ctrl (*p* < 0.05)	[173]
HepG2 cells	Ctrl, 0.55 mM fructose (FC1), 1 mM fructose (FC2), 1 mM fructose + 0.1 µM insulin (FC3)	48	nr	nr	Ellman	GSH: Lower in FC1 and FC2 vs. Ctrl (*p* < 0.05)	[174]
AML12 cells	Ctrl, PA	24	nr	nr	High-performance liquid chromatography-mass spectrometry (HPLC-MS)	GSH: Lower in palmitate vs. Ctrl (*p* < 0.05)	[144]
L02 cells	Ctrl, FFAs, FFAs + peonidin 3-O-glucoside (P3G)	24	nr	nr	Fluorimetric	GSH: Lower in FFAs vs. Ctrl and vs. treatment at the higher dose (*p* < 0.01)	[175]
HepG2 cells	Ctrl, FFAs, FFAs + zeaxanthin (ZEA), FFAs + Fer-1	24	nr	nr	Ellman	GSH: - Lower in FFAs vs. Ctrl (*p* < 0.05) and vs. FFAs + Fer-1 group - Recovery in all treatment groups	[176]
AML12 and HepG2 cells	Ctrl, FFAs, erastin (E), Fer-1	24	nr	nr	Ellman	GSH: Lower in FFAs and E vs. Ctrl (*p* < 0.01)	[147]
HepG2 cells	Ctrl, FFAs, FFAs + sulphasalazine (SAS), FFAs + melatonin (MEL)	nr	nr	nr	Ellman	GSH: Lower in FFAs and SAS vs. Ctrl (*p* < 0.0001) Recovery with the higher dose of MEL (*p* < 0.0001)	[148]
HepG2 cells	40% fetal bovine serum (FBS), FBS + FENO, FBS + S2 compound	48	nr	nr	Enzymatic recycling	GSH/GSSG and GSH: Lower in FBS vs. Ctrl and vs. treatments (*p* < 0.05)	[92]
HepG2 cells	Ctrl, FFAs, FFAs + arbutin (ARB)	24	nr	nr	Ellman	GSH: Lower in FFAs vs. FFAs + ARB (*p* < 0.05)	[151]
HepG2 cells	Ctrl, FFAs, FFAs + ALA	24	nr	nr	HPLC-UV	GSH: - Lower in FFAs vs. Ctrl (*p* < 0.01) - Recovery with all treatments (*p* < 0.05, *p* < 0.01)	[177]
HepG2 cells	Ctrl, FFAs, FFAs + MC3482 compound		nr	nr	HPLC-UV	GSH: Lower in FFAs vs. Ctrl and vs. FFAs + MC3482 (*p* < 0.0001)	[178]

GSH: glutathione; GSSG: oxidized glutathione; Ctrl: control group; nr: not reported; NEFAs: nonesterified fatty acids; OA: oleic acid; FENO: fenofibrate; t-BHP: tert-butylhydroperoxide; PA: palmitic acid; PHG: phloroglucinol; ALA: α-lipoic acid; NAC: N-acetylcysteine; LFP: *Lycii fructus* polysaccharide; FFAs: free fatty acids; M3G: malvidin-3-O-glucoside; MrGa: malvidin-3-O-galactoside; BSO: buthionine sulfoximine; H: hesperetin; PIC: picroside II; FE: fat emulsion; Fru: fructose; CA: carminic acid; XHY-1: xiaoheiyao; MCDM: methionine and cystine deficient media; CHO: cholesterol; FC1: 0.55 mM fructose; FC2: 1 mM fructose; FC3: 1 mM fructose + 0.1 µM insulin; HPLC-MS: high-performance liquid chromatography-mass spectrometry; P3G: peonidin 3-O-glucoside; ZEA: zeaxanthin; E: erastin; SAS: sulphasalazine; MEL: melatonin; ARB: arbutin; FBS: fetal bovine serum.

**Table 4:** Studies on LMW thiols involvement in models of MASLD.

Model	Diet/Supplements	Length of Study (Weeks)	Thiol Levels in MASLD Model	Thiol Levels in Normal Diet (ND)	Method	*p*-Values	Ref.
C57BL/6 mice	ND, methionine, and choline-deficient (MCD) diet, ND + PCB-126, MCD + PCB-126	14	Not reported (nr)	nr	High-performance liquid chromatography-mass spectrometry (HPLC-MS)	*Hepatic CysGly and Cys:* - No significance between MCD vs. ND - Higher in MCD vs. MCD + PCB126 group (*p* < 0.05)	[110]
C57BL/6J mice	ND, high-fat diet (HFD)	14	*Hepatic homocysteine (median, interquartile range):* HFD: 2.3 (2.1–2.7) μM/100 mg;	*Hepatic homocysteine (median and interquartile range):* ND: 2.4 (2.1–2.9) μM/100 mg	HPLC-MS	No significance between HFD and ND	[179]
nr	ND, high-fat and high-fructose diet (HFHF), HFHF + lupeol (L), HFHF + MET	8	*Hepatic free thiols, mean ± standard deviation* (nmol/mg protein): HFHF: 0.28 ± 0.02 HFHF + L: 3.6 ± 0.10 HFHF + MET: 3.3 ± 0.20	*Hepatic thiols, mean ± standard deviation* (nmol/mg protein): ND: 3.9 ± 0.30	Ellman	- Lower in HFHF vs. ND (*p* < 0.05) - Recovery with treatments (*p* < 0.05)	[180]

ND: normal diet; MCD: methionine-choline–deficient; nr: not reported; HPLC-MS: high-performance liquid chromatography-mass spectrometry; HFD: high-fat diet; HFHF: high-fat and high-fructose diet; L: lupeol; MET: metformin.

**Table 5:** Observational studies evaluating the levels of LMW thiols in human MASLD.

Age	Study Type	Patient Number	LMW Thiols	Method	*p*-Value	Ref.
Adults	Prospective observational (case-control)	Controls: 40 Patients with early MASLD: 29 Patients with advanced MASLD: 38	Plasma GSH, median, interquartile range (mg/mg protein): Control: 0.79 (0.67–1.28) Early MASLD: 1.37 (1.07–2.25) Advanced MASLD: 1.66 (1.08–2.02)	Ellman	Higher in early and advanced MASLD vs. controls (*p* < 0.001)	[182]
Adults	Retrospective, observational (population-based cohort)	FLI < 60: 3911 FLI ≥ 60: 1651	Free thiols, mean ± standard deviation (µmol/L/g protein): FLI < 60: 5.05 ± 0.99 FLI ≥ 60: 4.91 ± 1.02	Ellman	Significance between FLI > 60 and FLI < 60 estimated through multivariable logistic regression analyses (*p* < 0.001)	[183]
Adults	Prospective observational (case-control)	Controls: 69 MASLD: 144	nr	Gas chromatography-mass spectrometry	Blood GSH: Lower in MASLD vs. controls (*p* < 0.001)	[184]
Adults	Prospective observational (cross-sectional)	Controls: 25 MASLD: 60	Free thiols, mean ± standard deviation (µmol/L) Control: 11.70 ± 0.33 MASLD: 10.61 ± 0.13	Ellman	Lower in MASLD vs. controls (*p* < 0.05)	[185]
Children	Retrospective observational (longitudinal: Baseline (T0) and 12-month follow-up (T1))	MASLD: 24	Plasma tGSH, median, interquartile range (μmol/L): T0: 26.0 (20.5–38.5) T1: 31.5 (25.5–38.5) Plasma Hcy, mean, standard deviation (μmol/L): T0: 15.7 ± 4.1 T1: 21.1 ± 9.3	High-performance liquid chromatography-fluorescence detector (HPLC-FD)	Plasma tGSH: ns Plasma Hcy: Lower in T0 vs. T1 (*p* < 0.05)	[186]
Adults	Prospective observational (case-control)	Controls: 8 MASLD: 6	nr	Fluorimetric	Peripheral mononuclear cells GSH: Lower in MASLD vs. control (*p* < 0.05)	[187]

LMW: Low-molecular-weight; MASLD: metabolic dysfunction-associated steatotic liver disease; HPLC-FD: high-performance liquid chromatography-fluorescence detector; GSH: reduced glutathione; tGSH: total glutathione; Hcy: homocysteine; nr: not reported; FLI: Fatty Liver Index.

**Table 6:** Clinical trials on LMW thiols involvement in human MASLD.

Age	Study Design	Patient Number	GSH Levels in the Placebo Group	GSH Levels in the Treatment Group	Method	*p*-Value	Ref.
Children	- Randomized, double-blind placebo-controlled trial. - Two arms: the arm of placebo (PLA); and the arm of treatment with hydroxytyrosol and vitamin E (HXTE). - Time: Baseline (T0) and 4-month follow-up (T1).	PLA: 40 HXTE: 40	Plasma GSH, mean ± standard deviation (μM): T0: 29.6 ± 51.8 T1: 52.2 ± 85.0 Plasma GSSG, mean ± standard deviation (μM): T0: 1.5 ± 2.3 T1: 4.0 ± 4.9 Plasma GSH/GSSG, mean ± standard deviation: T0: 40.8 ± 61.1 T1: 132.9 ± 108.7	Plasma GSH, mean ± standard deviation (μM): T0: 47.1 ± 13.0 T1: 101.8 ± 43.8 Plasma GSSG, mean ± standard deviation (μM): T0: 1.9 ± 0.9 T1: 4.9 ± 2.8 Plasma GSSG/GSH, mean ± standard deviation: T0: 77.7 ± 80 T1: 189.5 ± 121.0	High-performance liquid chromatography (HPLC)-UV	GSH and GSH/GSSG: - Higher at T1 vs. T0 in PLA and in HXTE (*p* < 0.02) GSSG: Higher at T1 vs. T0 in HXTE (*p* < 0.001)	[188]
Adults	- Randomized, double-blind, placebo-controlled trial. - Three arms: the arm of PLA; the arm of treatment with a low dose of pinitol (P300 mg); and the arm with a high dose of pinitol (P500 mg). - Time: Baseline (T0) and 12-week follow-up (T1).	PLA: 30 P300: 30 P500: 30	Serum GSH, mean ± standard deviation (μM): T0: 198.3 ± 37.8 T1: 217.6 ± 37.8	Serum GSH, mean ± standard deviation (μM): P300 (T0): 248.9 ± 38.3 P300 (T1): 273.8 ± 39.8 P500 (T0): 158.0 ± 28.4 P500 (T1): 186.7 ± 28.8	Ellman	Not significant (ns)	[189]
Adults	- Open-label, parallel-group, randomized–controlled trial. - Two arms: the arm of standard management therapy (SMT); and the arm of treatment with SMT plus *H. pylori*-eradication therapy (HPET). - Time: Baseline (T0) and 24-week follow-up (T1).	SMT: 28 HPET: 36	–	Serum GSH, median, interquartile range (µg/mL): SMT (T0): 192.4 (151.4–213.1) SMT (T1): 196.7 (181.0–213.7) HPET (T0): 159.0 (143.2–183.1) HPET (T1): 188.5 (167.4–221.3)	Ellman	Higher at T1 vs. T0 in HPET (*p* < 0.05)	[190]
Children	- Randomized, crossover, one-side open trial. - Two arms: the arm of calorie-restricted regimen (RCR); and the arm of RCR plus supplement of lycopene-rich tomato juice (RCRT). - Time: Baseline (T0), 60-day crossover (T1), 60-day follow-up (T2).	RCR: 27 RCRT: 34	Not reported (nr)	nr	Enzymatic recycling	Serum GSH/GSSG and GSH: Higher in RCR and RCRT at T2 vs. T0 (*p* < 0.01) Blood GSSG: Lower in RCRT at T1 and T2 vs. T0 (*p* < 0.01)	[191]
Adults	- Randomized, double-blinded, placebo-controlled trial. - Two arms: the arm of PLA; the arm of treatment with VSL#3^®^ (VSL#3). - Time: Baseline (T0) and 10-week follow-up (T1).	PLA: 16 VSL#3: 19	Blood GSH/GSSG: T0: 20 ± 12 T1: 21 ± 9	Blood GSH/GSSG: T0: 22 ± 10 T1: 26 ± 13	Enzymatic recycling	ns	[192]
Children	- Single-blind, randomized, controlled, parallel dietary intervention. - Two arms: the arm with low-fat diet (LFD); and the arm of treatment with Mediterranean diet (MD). - Time: Baseline (T0) and 12-week follow-up (T1).	LFD: 22 MD: 22	–	Blood GSH, mean ± standard deviation (mg/L): LFD (T0): 82.0 ± 118.10 LFD (T1): 81.5 ± 100.68 MD (T0): 62.5 ± 97.53 MD (T1): 87.0 ± 39.29	Ellman	Higher in the MD group compared to LFD at T1 (*p* < 0.05)	[193]
Adults	- Clinical study with a single arm. - The arm of treatment with pioglitazone. - Time: Baseline (T0) and 3-month follow-up (T1).	MASLD: 37	–	Serum Thiols, mean ± standard deviation (µmol/L): T0: 181.26 ± 59.92 T1: 182.44 ± 54.06	Ellman	Higher in T1 vs. T0 (*p* < 0.015)	[194]
Adults	- Clinical study with a single arm. - Retrospective analysis based on the adherence to MD: low adherence (LA) group; high adherence (HA) group. - Time: Baseline (T0) and 24-month follow-up (T1).	LA: 20 HA: 20	–	GSH, mean ± standard deviation (mmol/10^9^ erythrocytes) LA (T0): 5.8 ± 2.7 LA (T1): 6.8 ± 1.9 HA (T0): 6.0 ± 2.3 HA (T1): 8.5 ± 3.2	Ellman	Higher in HA at T1 vs. T0 (*p* < 0.05); and HA at T1 vs. LA at T1 (*p* < 0.05)	[195]

GSH: reduced glutathione; PLA: placebo; HXTE: hydroxytyrosol and vitamin E; GSSG: oxidized glutathione; HPLC: high-performance liquid chromatography; MASLD: metabolic dysfunction-associated steatotic liver disease; ns: not significant; HPET: *H. pylori*-eradication therapy; RCR: calorie-restricted regimen; RCRT: RCR + tomato; VSL#3: lifestyle intervention plus VSL#3^®^; tGSH: total glutathione; MD: Mediterranean diet; LA: low adherence to MD; HA: high adherence to MD.

## Data Availability

Data is contained within the article and Supplementary Materials.

## Supplementary Materials

The following supporting information can be downloaded at: https://www.mdpi.com/article/10.3390/antiox13121461/s1. Table S1. Studies assessing the levels of GSH forms in rat models of MASLD. Table S2. Studies assessing the levels of GSH forms in mice models of MASLD. Table S3. Studies assessing the levels of GSH forms in other models of MASLD.
