# Supplementary material for: Alterations in Glutathione Redox Homeostasis in Metabolic Dysfunction-Associated Fatty Liver Disease: A Systematic Review
Source: Antioxidants (Basel). 2024 Nov 28;13(12):1461. doi: 10.3390/antiox13121461 (PMC11672975; doi:10.3390/antiox13121461)
Supplement: Supplementary file 1 [file antioxidants-13-01461-s001.zip › antioxidants-3300682-supplementary--resub.pdf]

SUPPLEMENTARY DATA

Alterations in glutathione redox homeostasis in metabolic dysfunction–associated fatty liver disease: a systematic review

Lucia Cesarini, Flavia Grignaffini, Anna Alisi, Anna Pastore

List of Tables:

Supplementary Table S1 page 2

Supplementary Table S2 page 7

Supplementary Table S3 page 13

Table S1. Studies assessing the levels of GSH forms in rat models of MASLD.

Strain	Diet	Study length (weeks)	GSH and GSSG levels in model(s)	GSH in normal diet (ND)	Method	p-values	Ref
Sprague–Dawley	ND, high-fat diet (HFD), HFD + Berberine (BRB)	8	Not reported explicitly (nr)	nr	Ellman	*Hepatic GSH:* Lower in HFD vs ND and vs HFD + BRB ($p < 0.05$)	[55]
Wistar	ND, HFD, HFD + betaine, HFD + choline, HFD + folic acid (FA)	14	nr	nr	Enzymatic recycling	*Serum forms:*<br>- Lower total glutathione (tGSH), GSH and GSH/GSSG in HFD vs ND ($p < 0.05$).<br>- Recovery of tGSH with all treatments ($p < 0.05$)<br>- Recovery of GSH and GSH/GSSG mainly with FA ($p < 0.05$)<br>- Recovery of GSSG with all treatments at the highest doses ($p < 0.05$)<br>*Hepatic forms:*<br>- Lower tGSH, GSH and GSH/GSSG in HFD vs ND ($p < 0.05$).<br>- Recovery of GSH mainly with choline ($p < 0.05$)<br>- Recovery of GSH/GSSG with all treatments ($p < 0.05$)<br>- Higher GSSG in HFD vs ND ($p < 0.05$)<br>- Recovery of GSSG with all treatments ($p < 0.05$)	[56]
Wistar	ND, high carbohydrate and high-fat diet (HCaHF), HCaHF + luteolin	12	nr	nr	Ellman	*Hepatic GSH:* Lower in HCaHF vs ND and vs higher doses of treatments ($p < 0.001$)	[57]
Not reported (nr)	ND, HFD	8	nr	nr	Ellman	*Hepatic GSH:* Lower in HFD rats vs ND ($p < 0.05$)	[58]
Sprague–Dawley	ND, HFD, HFD + sodium tanshinone IIA sulfonate (TAN)	16	nr	nr	Immunosorbent	*Plasma GSH:*<br>- Lower in HFD vs ND and vs TAN ($p < 0.05$)<br>- Recovery not significant (ns)<br>*Hepatic GSH:*<br>- Lower in HFD vs ND and vs TAN ($p < 0.05$)<br>- Recovery with treatment	[59]
Wistar	ND, HFD, Ethanol (EtOH), HFD + EtOH, HFD + 7,8-dihydroxyflavone (7,8-DHF), EtOH + 7,8-DHF, HFD + EtOH + 7,8-DHF	12	nr	nr	Ellman	*Hepatic GSH:*<br>-Lower in all disease models vs ND ($p < 0.001$)<br>-Recovery with all treatments vs each associated disease model ($p < 0.05$)	[60]
Sprague–Dawley	ND, high-fructose diet (HfruD), HFruD + nicotinamide	16	nr	nr	Enzymatic recycling	*Hepatic GSH/GSSG:*<br>-Lower in HFD vs ND (0.001)	[61]

						- Dose-dependent increase for all treatments (respectively $p < 0.05$, $p < 0.01$, $p < 0.001$)	
Sprague-Dawley	ND, HFD, ND + pyrvinium pamoate (PP), HFD + PP	16	nr	nr	Ellman	*Hepatic GSH:* Lower in HFD vs ND and treatment group ($p < 0.01$)	[62]
Wistar	ND, sodium acetate (SAT), streptozotocin (STZ)/nicotinamide (DAB), DAB + SAT	6	nr	nr	Enzymatic recycling	*Hepatic GSH:* Lower in DAB vs ND and SAT groups ($p < 0.05$)	[63]
Wistar	ND, high-cholesterol (HC) diet, HC + boiled/fried/grilled shrimp	8	nr	nr	Immunosorbent	*Hepatic GSH:*<br>- Lower for HC vs ND<br>- Recovery with boiled and grilled	[64]
Sprague-Dawley	ND, ND + exercise (EXC), HFD + EXC	18	nr	nr	Ellman	*Hepatic GSH:* Lower in HFD vs ND and vs HFD + EXC: ($p < 0.01$)	[65]
Wistar	ND, HFD, HFD + hesperetin	16	nr	nr	Ellman	*Hepatic GSH:* Lower in HFD vs ND and vs all treatments ($p < 0.05$)	[66]
Wistar	ND, Hypercaloric choline-deficient diet (HCCD), HCCD + α-lipoic acid (LA)	8	nr	nr	Enzymatic recycling	*Hepatic GSH:* HCCD, HCCD + LA: $p < 0.05$ (n=6) vs ND<br>*Hepatic GSH/GSSG:* HCCD + LA: $p < 0.05$ (n=6) vs ND	[67]
Sprague-Dawley	ND, HFD, HFD + resveratrol + Jiuzhuan Huangjing pills	12	nr	nr	Ellman	*Hepatic GSH:*<br>- Lower in HFD vs ND ($p < 0.05$)<br>- Recovery with treatments at higher doses ($p < 0.001$)	[68]
Wistar	ND, HFD, HFD + cumin	8	nr	nr	Ellman	*Plasma GSH:* Lower in HFD vs ND ($p < 0.001$)<br>*Hepatic GSH:* Lower in HFD vs ND and vs HFD + cumin ($p < 0.001$)	[69]
Sprague-Dawley	ND, HFD, HFD + AP39	7	nr	nr	Ellman	*Hepatic GSH:* Lower in HFD vs ND<br>Recovery with treatments ($p < 0.05$)	[70]
Wistar	ND, HFD + STZ, HFD + STZ + beetroot (BE)	5 (ND/HFD + STZ) + 12 (ND + no drug/HFD + STZ/HFD + STZ + BE)	nr	nr	Immunosorbent	*Hepatic GSH:* Lower in HFD + STZ vs ND ($p < 0.05$)<br>Recovery in treatments ($p < 0.05$)	[71]
Wistar	ND, HFD, HFD + policosanol, HFD + atorvastin, HFD + silymarin	8 (ND/HFD) + 8 (ND + no drug/HFD + policosanol, HFD + atorvastin, HFD + silymarin)	nr	nr	Ellman	*Hepatic GSH:*<br>- Lower in HFD vs ND ($p < 0.001$)<br>- Recovery with treatments ($p < 0.01$)	[72]
Wistar	ND, HFD, ND + zingiberene (ZB) HFD + ZB	15 (ND/HFD) + 6 (ND + no drug/HFD + ZB)	nr	nr	Ellman	*Hepatic GSH:* Lower in HFD vs ND and vs HFD + ZB ($p < 0.05$)	[73]

Sprague-Dawley	ND, HFD, ND + safranal (SAF), HFD + SAF	12	nr	nr	Ellman	*Hepatic GSH:*<br>Lower in HFD vs ND ($p < 0.001$) and vs SAF at higher dose ($p < 0.05$);	[74]
Wistar	ND, HFD, ND + astaxanthin (ASX), HFD + ASX, HFD + ASX + brusatol (B)	8	nr	nr	Fluorimetric	*Hepatic GSH:*<br>- Lower in HFD vs ND ($p < 0.05$)<br>- Lower in HFD + ASX vs HFD + ASX + B ($p < 0.05$)	[75]
Sprague–Dawley	ND, Fructose (F), F + *Lactobacillus acidophilus*, F + *Bifdobacterium* spp., F + *Bacillus coagulans*, F + *Lactobacillus rhamnosus*, F + mix	9	nr	nr	High-performance liquid chromatography (HPLC)-UV	*Hepatic GSH:*<br>- Lower in F vs ND ($p < 0.05$)<br>- Recovery with treatments ($p < 0.05$)	[76]
nr	ND, HFD + STZ, HFD + STZ + dapagliflozin (DP)	8 (ND/HFD + STZ) + 6 (ND/HFD + STZ/ HFD + STZ + DP)	nr	nr	Immunosorbent	*Hepatic GSH:*<br>- Lower in HFD + STZ vs ND ($p < 0.05$)<br>- Recovery with treatments ($p < 0.05$)	[77]
Sprague-Dawley	ND, HFD, HFD + Que Zui tea hot water extract, HFD + aqueous-ethanol extract	14	nr	nr	Ellman	*Hepatic GSH:*<br>- Lower in HFD vs ND ($p < 0.01$)<br>- Recovery with treatments ($p < 0.01$)	[78]
Sprague-Dawley	ND, HFD, HFD + Danshen zexie, HFD + Yishanfu	8	nr	nr	Ellman	*Hepatic GSH:*<br>- Lower in HFD vs ND ($p < 0.05$)<br>- Recovery with all treatments ($p < 0.05$)	[79]
nr	ND, HFD, HFD + fenofibrate (FENO), HFD + total flavonoids from *Scleromitron diffusum* (Ey-TFH)	3 (ND/HFD) + 2 (ND/HFD + no drug/HFD + Fe/HFD + Ey-TFH)	nr	nr	Ellman	*Hepatic GSH:*<br>Lower in HFD vs ND ($p < 0.01$)<br>Recovery with treatment at higher dose ($p < 0.05$)	[80]
Sprague-Dawley	ND, HFD, HFD + unacylated ghrelin	8	nr	nr	Fluorimetric	*Hepatic GSH:*<br>Lower in HFD vs ND ($p < 0.01$)<br>Recovery with all treatments ($p < 0.05$)	[81]
Wistar	ND, HFD, ND + zingerone (Z), HFD + Z, HFD + Z + compound C (CC)	12	nr	nr	Immunosorbent	*Hepatic GSH:*<br>Lower in HFD vs ND and vs HFD + Z ($p < 0.05$)	[82]
Wistar	ND, HFruD, HFruD + ellagic acid (EA), HFruD + allopurinol (A)	4 (ND/HFruD) + 4 (HFruD/ND + no drug/ HFruD + EA/ HFruD + A)	nr	nr	Immunosorbent	*Serum GSH:*<br>- Lower in HFruD vs ND ($P < 0.001$)<br>- Recovery with treatments ($p < 0.01$)	[83]
Wistar	ND, HFD, HFD + lizhong, HFD + vitamin E (VitE)	4 (ND/HFD) + 4 (ND + no drug/HFD/HFD + drugs)	nr	nr	Ellman	*Hepatic GSH:*<br>- Lower in HFD vs ND ($p < 0.05$)<br>- Recovery with all treatments ($p < 0.05$)	[84]
Wistar	ND, HFD, HFD + silymarin + HFD + metformin (MET), HFD + pioglitazone (PIO), HFD + dapagliflozin (DAPA), HFD + MET + PIO,	3 (ND/HFD) + 3 (ND + no drug/HFD + drugs)	nr	nr	Ellman	*Hepatic GSH:*<br>- Lower in HFD vs ND ($p < 0.05$)<br>- Recovery with all treatments ($p < 0.05$)	[85]

	HFD + MET + DAPA, HFD + PIO + DAPA						
Wistar	ND, HFD + STZ, HFD + STZ + Kavolì extract (KE)	4 (ND/HFD + STZ) + 5 (ND + no drug/HFD + STZ/HFD + STZ + KE	*Hepatic GSH (µmol/g tissue):* HFD + STZ: 1260.77 ± 444.05 HFD + STZ + KE: 1477.95 ± 813.02	*Hepatic GSH (µmol/g tissue):* 2059.50 ± 348.77	Fluorimetric	ns	[86]
Sprague–Dawley	ND, HFD, HFD + low, medium and intensive physical EXC	16 (ND/HFD + 6 (ND/HFD + EXC)	nr	nr	Ellman	*Hepatic GSH:*<br>- Lower in HFD vs ND ($p < 0.05$)<br>- Recovery with physica1 low and medium intensity EXC ($p < 0.05$)	[87]
Sprague-Dawley	ND, HFD + water extract from artichoke (WEA)	8 (ND/HFD) + 8 HFD + WEA)	nr	nr	Ellman	*Hepatic GSH:*<br>- Lower in HFD vs ND ($p < 0.05$)<br>- Recovery with all treatments ($p < 0.05$)	[88]
Wistar	ND, ND + N-Acetylcysteine (NAC), ND + γ-Oryzanol, HFD, HFD + NAC, HFD + γ-Oryzanol, HFD + NAC + γ-Oryzanol	17	nr	nr	Immunosorbent	*Hepatic GSH:*<br>- Lower in HFD vs ND groups ($p < 0.05$)<br>- Recovery with all treatments ($p < 0.05$)	[89]
Sprague Dawley	ND, high-fat choline-deficient diet (HFCD)	28	nr	nr	Fluorimetric	*Serum GSH:*<br>Lower in HFCD vs ND ($p < 0.001$)	[90]
Wistar	ND, ND + royal jelly (RJ), HFD, HFD + RJ, HFD + RJ + CC	16	nr	nr	Immunosorbent	*Hepatic GSH:*<br>Lower in HFD vs ND groups and vs NFD + RJ ($p < 0.001$)	[91]
Sprague–Dawley	ND, HFD, HFD + quinoa (Q)	12	nr	nr	Ellman	*Hepatic GSH:*<br>Lower in HFD vs ND and VS low quantity Q	[92]
Wistar	ND, ND + *Phaseolus vulgaris L.* bean leaves (BL), HFruD, HFruD + BL	13	nr	nr	Ellman	*Serum and hepatic GSH:*<br>Higher in HFruD vs ND	[93]
Zucker	ND + lard (C), C + fructooligosaccharides and raspberry polyphenolic extract (CFP)	4	nr	nr	Enzymatic recycling	*Hepatic GSH/GSSG:*<br>Higher in CFP vs C ($p < 0.05$)	[94]
Wistar	ND, ND + esculeogenin A (ESGA), HFD, HFD + ESGA	12	nr	nr	Immunosorbent	*Hepatic tGSH:*<br>- Lower in HFD vs ND ($p < 0.05$)<br>- Recovery with all treatments ($p < 0.05$)	[95]
Sprague–Dawley	ND, HFD, HFD + 4-butyl-polyhydroxybenzophenone, HFD + fenofibrate	12	nr	nr	Ellman	*Serum GSH:*<br>- Lower in HFD vs ND ($p < 0.05$)<br>- Recovery with treatments at higher doses ($p < 0.05$)	[96]

Wistar–Dawley	ND, ND + xanthohμmol (XH), HFD, HFD + XH, HFD + XH + CC	12	nr	nr	Immunosorbent	*Hepatic tGSH:* Lower in HFD vs ND and vs HFD + XH group ($p < 0.05$)	[97]
Wistar	ND, ND + topiramate (TPM), HFD, HFD + TPM	6 (ND/HFD) + 3 (ND + no drug/ND + TPM/HFD + TPM)	nr	nr	Ellman	*Hepatic GSH:* Lower in HFD and HFD + TPM vs ND ($p < 0.05$)	[98]
Sprague–Dawley	ND, ND + EtOH (model), model + Tiaogan Jiejiu Tongluo Formula, model + silybin	8	nr	nr	Ellman	*Hepatic GSH:* - Lower in model vs ND ($p < 0.001$) - Recovery with treatments ($p < 0.05$)	[99]
Sprague–Dawley	ND, HFD, HFD + lauric Acid	8	nr	nr	Ellman	*Hepatic GSH:* - Lower in HFD vs ND ($p < 0.05$) - Recovery with treatments ($p < 0.05$)	[100]
ZDF rats	ND, HFD, HFD + Tenovin-1	10 (ND/HFD) + 10 (ND + no drug/HFD/HFD + drug)	nr	nr	Enzymatic recycling	*Hepatic tGSH:* Lower in HFD vs ND ($p < 0.05$)	[101]
Sprague–Dawley	ND, HFCD, HFCD + VitE, HFCD + ornithine aspartate	16 (ND/HFD) + 12 (ND + no drug/HFD/HFD + drugs)	nr	nr	Fluorimetric	*Hepatic tGSH:* - Lower in HFCD vs ND ($p < 0.001$) - Recovery with treatments ($p < 0.001$)	[102]

GSH: glutathione; GSSG: oxidized glutathione; ND: normal diet; HFD: high-fat diet; BRB: berberine; nr: not reported explicitly; tGSH: total glutathione; FA: folic acid ; HCaHF: high carbohydrate and high-fat diet; TAN: sodium tanshinone IIA sulfonate; ns: not significant; EtOH: Ethanol; 7,8-DHF: 7,8-dihydroxyflavone; HfruD: high-fructose diet; PP: pyrvinium pamoate; SAT: sodium acetate; STZ: streptozotocin; DAB: streptozotocin /nicotinamide; HC: high-cholesterol; 1,25(OH)2D3: 1,25-dihydroxyvitamin D3; EXC: exercise; HCCD: hypercaloric choline-deficient diet; LA: α-lipoic acid; BE: beetroot; ZB: zingiberene; SAF: safranal; ASX: astaxanthin; B: brusatol; F: Fructose; HPCL: high-performance liquid chromatography; DP: dapagliflozin; DZD: Danshen zexie; FENO: fenofibrate; Ey-TFH: total flavonoids from *Scleromitron diffusum*; Z: zingerone; CC: compound C; EA: ellagic acid; A: HFruD + allopurinol; VitE: vitamin E; MET: metformin; PIO: pioglitazone; DAPA: dapagliflozin; KE: Kavolì extract; WEA: water extract from artichoke; NAC: N-Acetylcysteine; HFCD: high-fat choline-deficient diet; RJ: royal jelly; Q: quinoa; BL: *Phaseolus vulgaris L.* bean leaves; C: normal diet + lard; CFP: normal diet + lard + fructooligosaccharides and raspberry polyphenolic extract; ESGA: esculeogenin A; XH: xanthohμmol; TPM: topiramate; model: normal diet + EtOH.

Table S2. Studies assessing the levels of GSH forms in mice models of MASLD.

Strain	Diet	Study length (weeks)	GSH and GSSG levels in model(s)	GSH in normal diet (ND)	Method	p-values	Ref
C57BL/6J	ND, methionine, and choline-deficient diet (MCD), MCD + leonurine hydrochloride (LH)	6	nr	nr	Ellman	*Hepatic GSH:* Lower in MCD vs ND and vs MCD + LH at higher dose ($p < 0.05$)	[109]
C57BL/6	ND, ND + PCB-126, MCD, MCD + PCB-126	4 (ND/ high-fat diet (HFD)) + 10 (ND + no drug/ND + PCB-126/HFD + PCB-126)	nr	nr	High-performance liquid chromatography - mass spectrometry (HPLC-MS)	*Hepatic GSH:* - No significance between MCD vs ND - Higher in MCD vs MCD + PCB126 group ($p < 0.05$)	[110]
C57BL/6	ND, MCD, MCD + betaine (BET)	6	nr	nr	Ellman	*Hepatic GSH:* - Lower in MCD vs ND and vs MCD + BET ($p < 0.05$)	[111]
IDH2 KO and WT C57BL/6	ND, HFD	16	nr	nr	Enzymatic recycling	*Hepatic mito- and cyto-GSSG/tGSH:* - Higher in HFD vs ND ($p < 0.05$) - Higher in IDH2 KO HFD vs HFD WT ($p < 0.05$) vs WT HFD	[112]
Swiss	ND, ND + açai, HFD, HFD + açai	6 (ND/HFD) + 6 (ND + no drug/HFD + açai)	nr	nr	Enzymatic recycling	*Hepatic tGSH:* - Increases in HFD and treatment group vs ND ($p < 0.05$) *Hepatic GSSG:* Increases in HFD vs ND	[113]
C57BL/6J	ND, HDF	10	nr	nr	Fluorimetric	*Hepatic tGSH, GSH/GSSGS:* Lower in HFD vs ND ($p < 0.05$) *GSSG:* Higher in HFD vs ND ($p < 0.05$)	[114]
C57BL/6	ND, HFD, HFD + indole-3-acetic acid (IAA)	12	nr	nr	Enzymatic recycling	*Hepatic GSH, GSH/GSSG:* Lower in HFD vs ND and vs HFD + IAA ($p < 0.05$)	[115]
ICR	ND, HFD, HFD + chinese herbal formula (CHF03)	8	nr	nr	Ellman	*Hepatic GSH:* Lower in HFD vs ND ($p < 0.01$) and vs HFD + CHF03 ($p < 0.05$)	[116]

Lepdb/Lepdb (db/db) and Lepdb/m (db/m) C57BLKS/J	ND, ND + quercetin (QUR)	8	nr	nr	Ellman	*Hepatic GSH:* Lower in db/db + ND vs db/m + ND ($p < 0.05$) and vs db/db + ND + QUR ($p < 0.01$)	[117]
C57BL/6	ND, MCD, MCD + deferoxamine mesylate salt (DFO), MCD + DFO + RSL-3	24	nr	nr	Enzymatic recycling	*Hepatic GSH:* - Lower in MCD vs ND ($p < 0.05$) - Lower in MCD vs MCD + RSL-3 and MCD + DFO + RSL-3 ($p < 0.05$)	[118]
C57BL/6	High fat high sucrose (HFHS) diet + *Codonopsis lanceolata* polysaccharide (CLPS)	8	nr	nr	Enzymatic recycling	*Hepatic GSH:* Lower in HFHS vs ND and vs HFHS + CLPS ($p < 0.05$)	[119]
C57BL/6J	ND, ND + *Platycodon grandiflorus* (PG), HFD, HFD + PG	16	nr	nr	Fluorimetric	*Hepatic tGSH:* - Lower in HFD vs ND and vs HFD + PG ($p < 0.05$)	[120]
MAT1A siGls1/siCtrl C57BL/6	MAT1A KO siGls1/siCtrl + HFCD, KO siGls1/siCtrl + MCD	3 (ND/HFCD/MCD) + 3 (ND + no drug/HFCD + drugs/MCD + drugs)	nr	nr	HPLC-MS	*Hepatic GSSG/GSH:* - Higher in MCD + siCtrl vs ND and MCD + siGls1 ($p < 0.05$) - Lower in HFCD + siGls1 vs HFCD + siCtrl ($p < 0.05$)	[121]
DUSP7 KO C57BL/6	ND, HFD	16	nr	nr	Ellman	*Hepatic GSH:* - Lower in HFD WT vs ND WT ($p < 0.05$) - Lower in HFD DUSP7 KO vs HFD WT ($p < 0.05$)	[122]
nr	ND, HFD	4	nr	nr	Enzymatic recycling	*Hepatic GSH:* Lower in HFD vs ND ($p<0.05$)	[123]
C57BL/6	HFD + streptozotocin (STZ), HFD + STZ + *Garcinia indica* extract (GIE), HFD + STZ + curcuminoids (CUR), HFD + STZ + GIE + CUR	4 (HFD + STZ) + 4 (ND + no drug/HFD + STZ + drugs)	nr	nr	Ellman	*Hepatic GSH:* Recovery in treatments vs HFD + STZ ($p < 0.01$)	[124]
C57BL/6J	ND, HFD + ethanol (EtOH)	12	nr	nr	Enzymatic recycling	*Hepatic GSSG/GSH:* Lower in HFD + EtOH vs ND ($p < 0.05$)	[125]

C57BL/6	ND, HFD, HFD + neohesperidin (NHP)	12	nr	nr	Ellman	*Hepatic GSH:* Lower in HFD vs ND and vs HFD + NHP ($p < 0.05$)	[126]
C57BL/6	ND, ND + phoenixin 14 (P14), HFD, HFD + P14	10	nr	nr	Fluorimetric	*Hepatic GSH:* Lower in HFD vs ND and VS HFD + P14 ($p < 0.01$)	[127]
C57BL/6	ND, HFD, HFD + resveratrol (RSV), HFD + RSV + *Bifidobacteria longum* (BL)	8	nr	nr	Ellman	*Hepatic GSH:* - Lower in HFD vs ND ($p < 0.001$) Recovery with all treatments ($p < 0.05$)	[128]
ICR	High- fat high-fructose (HFHF) + korean black ginseng (BG)	Induction period (ND/HFHF) + 8 (ND/HFHF + BG)	nr	nr	Immunosorbent	*Hepatic GSH:* - Lower in HFHF ns ND ($p < 0.05$) - Recovery with all treatments ($p < 0.05$)	[129]
C57BL/6	Choline-deficient, L-amino acid-defined, high-fat diet (CDAAH), CHAAH + aloin	12	nr	nr	Enzymatic recycling	*Hepatic GSH:* Lower in CDAAH vs ND and vs CDAAH + aloin ($p < 0.01$)	[130]
C57BL/6J	ND, HFD, HFD + RSV	8 (ND/HFD) + 4 (ND + no drug/HFD + RSV)	nr	nr	Enzymatic recycling	*Hepatic GSH:* Lower in HFD vs ND ($p < 0.01$) and vs HFD + RSV ($p < 0.001$) *Hepatic GSH/GSSG:* Lower in HFD vs ND ($p < 0.01$) and vs HFD + RSV ($p < 0.01$)	[131]
C57BL/6	ND, ND + trelagliptin (TR), HFD, HFD + TR	10	nr	nr	Ellman	*Hepatic GSH:* Lower in HFD vs ND ($p < 0.001$) and vs HFD + TR ($p < 0.01$)	[132]
C57BL/6J	ND, HFD, HFD + dehydroabietic acid (DA)	3 (ND/HFD) + 9 (ND + no drug/HFD + DA)	nr	nr	Ellman	*Hepatic GSH:* Lower in HFD vs ND ($p < 0.001$) and vs HFD + DA at higher dose ($p < 0.05$)	[133]
Kunming	ND, HFHS, HFHS + berberine and curcumin combination via dextran-coated bilosomes (DEAE-DEX@LSDBC)	8	nr	nr	Ellman	*Hepatic GSH:* Lower in HFHS vs ND ($p < 0.001$) and DEAE-DEX@LSDBC ($p < 0.01$)	[134]

C57BL/6	ND, ND + decabromodiphenyl ether (BDE209), HFD, HFD + BDE209	8	nr	nr	Immunosorbent	*Hepatic GSH:* Lower in HFD and HFD + BDE209 vs ND ($p < 0.05$)	[135]
C57BL/6J	ND, HFD, HFD + oleuropein (Ole)	8 (ND/HFD) + 8 (ND + no drug/HFD + Ole	nr	nr	Ellman's method	*Blood tGSH/GSSG:* Lower in HFD vs ND ($p < 0.001$)	[136]
C57BL/6	ND, HFD, HFD + melatonin (MEL)	4 (ND + MEL) + 24 (ND/HFD + no drug, HFD + MEL)	nr	nr	Ellman	*Hepatic GSH:* -Lower in HFD vs ND ($p < 0.0001$) - Recovery with all treatments ($p < 0.001$)	[137]
C57BL/6J	ND, HFD, HFD + *Ramulus Mori* (Sangzhi) alkaloids (SZ-A)	14 (ND/HFD) + 6 (ND + no drug/HFD + SZ-A)	nr	nr	Ellman	*Hepatic GSH:* - HFD vs ND not significant - Higher in HFD + SZ-A vs HFD	[138]
PTEN KO C57BL/6J	Probiotics	8 / 40 endpoints	nr	nr	Enzymatic recycling	*Hepatic tGSH:* Lower in PTEN KO vs WT and vs PTEN + probiotics ($p < 0.05$)	[139]
C57BL/6J	ND, HFD, HFD + *Poria cocos* polysaccharides (PCP)	4 (HD/HFD) + 8 (ND + no drug/HFD + PCP)	nr	nr	Ellman	*Hepatic GSH:* Lower in HFD vs ND and HFD + PCP at higher dose ($p < 0.05$)	[140]
C57BL/6J	ND, MCD, high fat and high cholesterol diet (HFHC), MCD + hUC-MSCs exosomes (EXO), HFHC + EXO	20	nr	nr	Ellman	*Hepatic GSH:* Lower in MCD and HFHC vs ND and vs MCD + EXO and HFHC + EXO ($p < 0.05$)	[141]
Ob/ob, strain nr	ND, Amylin liver NASH diet (AMLN), AMLN + obeticholic acid (OCA), AMLN + Ferrostatin-1 (FT1), AMLN + OCA + FT1	24 (ND/AMLN) + 7 (ND + no drug/AMLN + drugs)	nr	nr	Immunosorbent	*Hepatic GSH*: Lower in AMLN vs ND ($p < 0.05$)	[142]
C57BL/6	ND, HFD, HFD + acacetin (Aca)	16	nr	nr	Ellman	*Hepatic GSH:* Lower in HFD vs ND and vs HFD + Aca at medium dose ($p < 0.05$)	[143]
C57BL/6J-OlaHsd	ND, ND + acetaminophen (APAP), western diet (WD), WD + APAP, WD + APAP + glycine (Gly)	10	nr	nr	HPLC-MS	*Hepatic GSH/GSSG:* Lower in WD + APAP vs ND + APAP ($p < 0.05$) *Hepatic GSH:* Lower in WD + APAP vs WD ($p < 0.001$) and vs WD + APAP + Gly ($p < 0.01$)	[144]

$ApoE^{-/-}$, strain nr	HFD, HFD + silica nanoparticles (SiNPs)	4 (HFD) + 12 (HFD/HFD + drugs)	nr	nr	Enzymatic recycling	*Hepatic GSSG:* Higher in HFD + SiNPs at higher dose vs HFD ($p < 0.05$) *Hepatic GSH/GSSG:* Lower in HFD + SiNPs at higher doses vs HFD ($p < 0.05$)	[145]
C57BL/6J	HFD + atractylodin (Atr)	8 (ND/HFD) + 8 (ND + no drug (HFD + Atr)	nr	nr	Ellman	*Hepatic GSH:* - Lower in HFD vs ND ($p < 0.001$) - Recovery at higher doses of treatment ($p < 0.01$)	[146]
Hepatocyte conditional KO (HO-1) HEPKO C57BL/6J	ND, HFD	32	nr	nr	Ellman	*Hepatic GSH:* - Lower in HFD vs ND ($p < 0.05$) - Lower in HFD + HO-1 HEPKO vs HFD ($p < 0.05$)	[147]
C57BL/6	ND, HFD, HFD + MEL	8	nr	nr	Ellman	*Hepatic GSH:* - Lower in HFD vs ND ($p < 0.0001$) - Recovery in all treatments ($p < 0.05$)	[148]
Low-density lipoprotein receptor (Ldlr) KO C57BL/6J	WD, low fat diet (LFD)	1, 4, 8, 20, 40 endpoints	nr	nr	HPLC-MS	*Hepatic GSH/GSSG:* Higher in WD 4 wk, WD 8 wk vs LFD ($p < 0.05$)	[149]
C57BL/6	ND, HFD, HFD + APAP	8	nr	nr	Ellman	*Hepatic GSH:* - Lower in HFD vs ND ($p < 0.01$) - Lower in HFD + APAP vs HFD ($p < 0.01$)	[150]
C57BL/6	ND, HFD, HFD + arbutin (ARB)	10	nr	nr	Ellman	*Hepatic GSH:* Lower in HFD vs ND ($p < 0.01$) and vs HFD + ARB ($p < 0.05$)	[151]
C57BL/6	ND, MCD, MCD + naringenin (Nar)	6	nr	nr	Ellman	*Hepatic GSH:* Lower in MCD vs ND and vs MCD + Nar ($p < 0.05$)	[152]
PLIN5 KO C57BL/6J	ND, high fructose diet	20	nr	nr	Ellman	*Hepatic GSH:* - Lower in HFD vs ND ($p < 0.001$) - Higher in PLIN5 KO + HFD vs HFD ($p < 0.001$)	[153]
C57BL/6J	ND, MCD, MCD + 6-shogaol (6S)	4	nr	nr	Enzymatic recycling	*Hepatic GSH:* Lower in MCD vs ND ($p < 0.001$) and vs MCD + 6S ($p < 0.05$)	[154]
db/db C57BLKS/J	ND, ND + MET, ND + Ras-selective lethal 3 (RSL3) + MET	6	nr	nr	Enzymatic recycling	*Hepatic tGSH/GSSG:* - Lower in db/db vs ND and vs db/db + MET ($p < 0.05$) - Lower in db/db + MET + RSL3 vs db/db + MET and higher than db/db ($p < 0.05$)	[155]

C57BL/6 J	ND, HFD + CCl_4, HFD + CCl_4 + silymarin, HFD + CCl_4 + *Alisma orientale* extract (AE)	10 (ND/HFD) + 4 (ND + no drug/HFD + drugs)	nr	nr	Ellman	*Hepatic GSH:* Lower in HFD + CCl_4 vs ND ($p < 0.01$) and vs HFD + CCl_4 + AE at higher dose ($p < 0.001$)	[156]

GSH: glutathione; GSSG: oxidized glutathione; ND: normal diet; MCD: methionine, and choline-deficient diet; LH: leonurine hydrochloride; HFD: high-fat diet; HPCL: high-performance liquid chromatography; BET: betaine; IAA: indole-3-acetic acid; CHF03: chinese herbal formula; QUR: quercetin; DFO: deferoxamine mesylate salt; HFHS: high fat high sucrose diet; CLPS: *Codonopsis lanceolata* polysaccharide; PG: *Platycodon grandiflorus*; tGSH: total glutathione; STZ: streptozotocin; GIE: *Garcinia indica* extract; CUR: curcuminoids; EtOH: ethanol; NHP: neohesperidin; P14: phoenixin 14; RSV: resveratrol; BL: *Bifidobacteria longum*; BG: korean black ginseng; HFHF: high-fat high-fructose; CDAAH: Choline-deficient, L-amino acid-defined, high-fat diet; TR: trelagliptin; DA: dehydroabietic acid; DEAE-DEX@LSDBC: berberine and curcumin combination via dextran-coated bilosomes; BDE209: decabromodiphenyl ether; Ole: oleuropein; MEL: melatonin; SZ-A: *Ramulus Mori* (Sangzhi) alkaloids; PCP: *Poria cocos* polysaccharides; HFHC: high fat and high cholesterol diet; EXO: hUC-MSCs exosomes; AMLN: Amylin liver NASH diet; OCA: obeticholic acid; FT1: Ferrostatin-1; Aca: acacetin; APAP: acetaminophen; WD: western diet; Gly: glycine; SiNPs: silica nanoparticles; Atr: atractylodin; HO-1: Hepatocyte conditional KO; Ldlr: Low-density lipoprotein receptor; LFD: low fat diet; wk: week; ARB: arbutin; Nar: naringenin; 6S: 6-shogaol; RSL3: Ras-selective lethal 3; AE: *Alisma orientale* extract.

Table S3. Studies assessing the levels of GSH forms in other models of MASLD.

Model	Diet/treatments	Study length	GSH levels in model(s)	GSH in normal diet (ND)	GSH assay method	P-values	Ref
AB strain zebrafish (Danio rerio) and Tg (fabp10a: dsRed) zebrafish	ND, high cholesterol diet (HCD), HCD + berberine (BBR)	10 days	nr	nr	Ellman	*Hepatic GSH:* Lower in HCD vs ND and HCD + BBR ($p < 0.05$)	[157]
WT zebrafish (AB strain), liver-specific eGFP transgenic zebrafish (Tg (lfabp10α:eGFP)), macrophage-specific red fluorescent protein expression transgenic line (Tg (mpeg1:dsred))	ND, ND + thioacetamide (TAA), ND + TAA + limonin	72 hours	nr	nr	Fluorimetric	*Hepatic GSH:* - Lower in TAA vs ND and vs TAA + limonin ($p < 0.001$)	[158]
New Zealand White rabbits	ND, high-fat diet (HFD), HFD + *M. peregrine* (MP), HFD + simvastatin (SIM)	16 weeks	*Serum GSH (µmol/mL):* HFD: 35.12 HFD + MP: 49.37 HFD + SIM: 61.38	*Serum GSH (µmol/mL):* 71.50	Ellman	- Lower in HFD vs ND ($p < 0.05$) - Recovery with treatments ($p < 0.05$)	[159]
Cynomolgus monkeys	NASH diet (NASHD), NASHD + DT-109	40 weeks (NASHD) + 20 weeks (NASHD + DT-109)	nr	nr	Fluorimetric	*Hepatic GSH:* - Higher in treatment vs NASH diet ($p < 0.01$)	[160]

GSH: glutathione; ND: normal diet; HCD: high cholesterol diet; BBR: berberine; TAA: thioacetamide; HFD: high-fat diet; MP: *M. peregrine*; SIM: simvastatin; NASHD: NASH diet.
